# A management of patients achieving clinical complete response after neoadjuvant therapy and perspectives: on locally advanced rectal cancer

**DOI:** 10.3389/fonc.2024.1450994

**Published:** 2025-01-08

**Authors:** Yu-Xin Liu, Xin-Rong Yang, Lan-Qing Peng, Zhuo-Hong Li

**Affiliations:** Department of Oncology, Hospital of Chengdu University of Traditional Chinese Medicine, Chengdu, Sichuan, China

**Keywords:** locally advanced rectal cancer, neoadjuvant treatment, clinical complete response, watch and wait policy, organ preservation

## Abstract

Neoadjuvant chemoradiotherapy (nCRT) followed by total mesorectal excision (TME) and selective use of adjuvant chemotherapy is currently considered the standard of care for locally advanced rectal cancer (LARC). Despite this, the concept of organ preservation is gradually challenging this approach. The management of complete clinical remission (cCR) lacks international consensus, leading scholars to develop their own perspectives based on well-designed studies and long-term data from large multicenter cohorts. To ensure appropriate treatment, this review focuses on the choice of neoadjuvant therapy, criteria for defining cCR, and treatment strategies for patients who achieve cCR after neoadjuvant therapy. By providing guidance on the accurate management of LARC patients after cCR, this review aims to prevent over- or under-treatment.

## 1 Introduction

For Colorectal cancer is the third most prevalent cancer in the world, and rectal cancer accounts for approximately 31% of all cases (1, 2). locally advanced rectal cancer (LARC) is typically defined as stages II-III of the disease. The current standard treatment for advanced rectal cancer is total mesorectal excision (TME) followed by preoperative chemoradiotherapy, as supported by the Dutch trial and the German trial CAO/ARO/AIO-94 (3). In recent years, there has been a tendency to increase the intensity of systemic chemotherapy both before and after preoperative radiotherapy (4–6). Moreover, the introduction of total neoadjuvant therapy (TNT) has further improved the rate of achieving complete pathological response (pCR) in advanced rectal cancer patients after neoadjuvant therapy (7–10). Notably, pCR can only be determined through examination of resected tissue after surgery (11). However, for patients who have achieved complete tumor regression after preoperative neoadjuvant therapy, the necessity of surgery remains uncertain.

In 2009, Brazil’s Habr-Gama proposed the “watch and wait method” (W&W) based on complete clinical response (12). Clinical complete remission refers (cCR) to the absence of evidence of residual tumor in the local area of the primary lesion confirmed by physical examination and auxiliary examination after neoadjuvant therapy (ycT0N0) (13). A meta-analysis comparing TME to the W&W strategy found similar survival outcomes in rectal cancer patients who achieved cCR after neoadjuvant radiotherapy, suggesting that patients achieving cCR after neoadjuvant therapy, surgical intervention may not be the only course of action.

Amidst the excitement, a recent study has indicated that the correlation between cCR and pCR is still unsatisfactory, mainly due to the limitations of existing diagnostic methods. The criteria may be too lenient, including patients who have not reached the “safe range” in the W&W cohort, or too strict, leading to radical surgery (RS) for patients who might otherwise be evaluated for complete clinical response (14–16). Furthermore, opting for the W&W approach poses a potential risk of residual tumors for patients (17–20). Research indicates that around 20–30% of patients who reach cCR after neoadjuvant chemoradiotherapy (nCRT) could experience local tumor regrowth during the monitoring period (21). This prompts us to question whether the decision to pursue the W&W strategy is appropriate for individuals who achieve cCR following neoadjuvant therapy.

**Figure 1:**
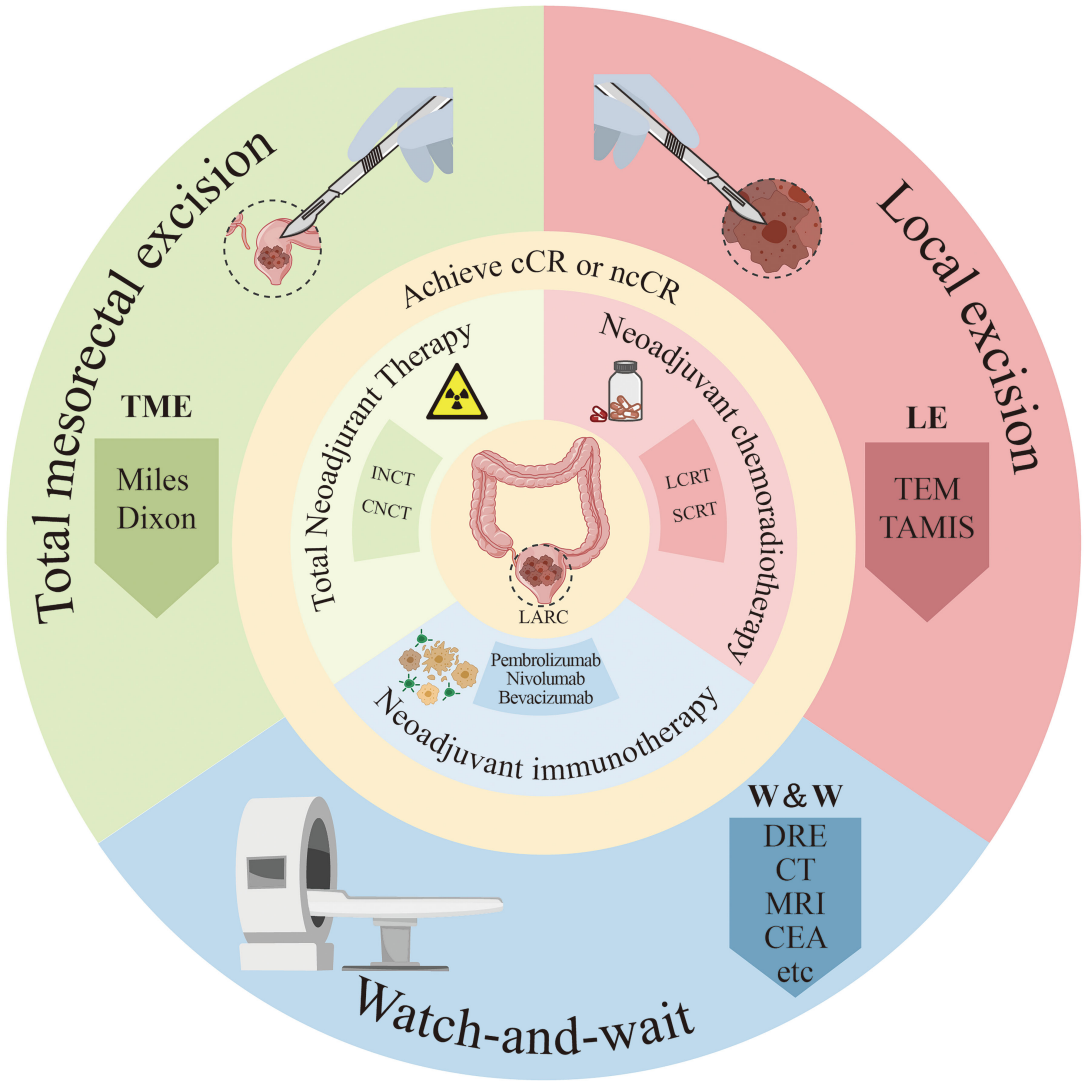
Diagram of treatment methods for locally advanced rectal cancer patients. ncCR, near-complete response; cCR, clinical complete response; SCRT, short-course radiotherapy; LCRT, long course concurrent chemoradiation; INCT, induction chemotherapy; CNCT, consolidation chemotherapy; DRE, digital rectal examination; CEA, Serum carcinoembryonic antigen; Miles, abdominoperineal resection; Dixon, low anterior resection; TEM, traditional transanal endoscopic microsurgery; TAMIS, transanal minimally invasive surgery.

The objective of this article is to provide a comprehensive overview of therapeutic strategies for patients who have achieved cCR following neoadjuvant therapy, including the establishment of cCR criteria and methods for monitoring recurrence (**Figure 1**). Additionally, we seek to review the latest advancements in neoadjuvant therapy for advanced rectal cancer. By analyzing clinical trials, we intend to assess both non-surgical and surgical options and propose diagnostic and treatment approaches for patients with cCR following neoadjuvant therapy.

## 2 Current status of neoadjuvant therapy for advanced rectal cancer

Preoperative chemoradiotherapy has become the standard treatment for LARC since the mid-2000s. Until recently there are few studies have demonstrated the survival benefits and potential for improved pCR associated with preoperative chemoradiotherapy (22–26). Given the concept of non-operative management, researchers have conducted several investigations to enhance preoperative treatment options, such as TNT, short-course radiotherapy (SCRT), and long-course chemoradiotherapy (LCRT).

However, comparing the 2017 European Society for Medical Oncology (ESMO) guidelines and the 2022 National Comprehensive Cancer Network (NCCN) guidelines (27, 28). The selection of neoadjuvant therapy and the decision regarding chemotherapy before and after radiotherapy for rectal cancer remain subjects of controversy. Presently, efforts are being directed towards refining the TNT approach, investigating the intensity of chemotherapy to enhance response rates, and expanding the options for non-surgical and local surgical methods. This progressive direction focuses on optimizing TNT and exploring various strategies to improve treatment outcomes in rectal cancer patients.

### 2.1 Neoadjuvant chemoradiotherapy

Neoadjuvant therapy is a common treatment approach for individuals with LARC. Typically, this involves LCRT or SCRT. The chemotherapeutic agents fluorouracil or capecitabine are commonly used in the neoadjuvant setting (29, 30). While the standard strategy has resulted in a reduction in locoregional recurrence rates, improvements are still needed in terms of achieving a pCR and systemic disease control. In recent years, a series of trials exploring improved neoadjuvant treatment for LARC have shown that the addition of irinotecan to capecitabine based chemoradiotherapy before surgery can improve the pCR rate of patients with specific genetic markers (UGT1A1) (31); On the basis of radiotherapy, mfolfox6 (a chemotherapy regimen consisting of leucovorin, 5-FU and oxaliplatin) can improve the pCR rate of patients compared with the traditional fluorouracil regimen. Still, it does not significantly improve the 3-year disease free survival (DFS) (32). However, larger scale tests are still needed to verify the existing findings.

The conventional treatment plan for classical SCRT involves administering a dose of 5×5Gy, once a day, with each session delivering 5Gy over 5 consecutive days. Additionally, in classic SCRT, several radiotherapy techniques can be used to assist in treatment. For SCRT, 3D-CRT usually meets the treatment requirements, especially in situations where technical conditions are limited (33). However, IMRT (including VMAT) is more suitable for patients who need to protect surrounding normal tissues due to its precise dose control and reduced side effects. Therefore, using IMRT/VMAT would be a better choice, but it also depends on the availability of equipment and technology at the treatment center (34). Nonetheless, it is not advised to combine concurrent chemotherapy and targeted drugs (35, 36). In the Trans-Tasman Radiation Oncology Group (TROG) clinical trial, patients with T3 rectal cancer were randomized to receive either SCRT or LCRT before surgery. The study found that the pCR rate was significantly lower in the SCRT group (0.7%) compared to the LCRT group (16%). Additionally, the rate of positive circumferential resection margin (CRM) was higher in the SCRT group (12.9%) than in the LCRT group (4.4%) (37). This is primarily due to the short interval between radiotherapy and surgery, which limited tumor regression, and the absence of concurrent chemotherapy, which would have enhanced radiosensitivity and tumor response. Several trials have proved that the improved method is to carry out consolidation chemotherapy during the 4-8 week rest period between short-term radiotherapy and surgery, for it helps target and eradicate microscopic metastatic disease that may not be addressed by localized radiotherapy alone, further reducing tumor size and extent, increasing the likelihood of achieving negative surgical margins and improving pCR rates, and prevent tumor progression during the waiting period (38), thereby increasing the likelihood of achieving negative surgical margins and improving overall treatment outcomes.

### 2.2 Total neoadjuvant therapy

TNT refers to the transfer of all or part of adjuvant chemotherapy from the postoperative stage to the preoperative stage on the basis of the standard diagnosis and treatment scheme of nCRT + TME + postoperative adjuvant chemotherapy. Compared with the previous standard mode, this strategy has better pCR, downstaging rate, and lower recurrence rate and can avoid the effect of adjuvant chemotherapy affected by patient compliance and postoperative complications (39–52).

A recent meta-analysis comparing outcomes in patients with LARC who received TNT vs. Concurrent chemoradiotherapy followed by surgery and adjuvant chemotherapy (CRT plus A) (52). The results revealed that the pooled prevalence of pCR was 29.9% (range, 17.2%-38.5%; median, 27.7%) in the TNT group and 14.9% (range, 4.2%-21.3%; median, 13.8%) in the CRT plus A group. TNT was associated with a higher chance of achieving a pCR (odds ratio [OR], 2.44; 95% CI, 1.99-2.98; *P* < 0.001) and significantly higher odds of improved disease-free survival in patients who received TNT (OR, 2.07; 95% CI, 1.20-3.56; I^2^ = 49%; *P* =0.009). According to the different sequences of CRT and neoadjuvant chemotherapy, induction chemotherapy (INCT) with systemic chemotherapy given before CRT and consolidation chemotherapy (CNCT) with systemic chemotherapy given after CRT have been explored. Both methods showed improved results compared with conventional preoperative. Significantly, two prospective clinical trials, namely OPRA and CAO/ARO/AIO-12, have specifically investigated the sequencing of chemotherapy and concurrent chemoradiotherapy in rectal cancer patients.

The OPRA trial demonstrated comparable three-year disease-free survival (DFS) rates in both groups. However, compared with the INCT-CRT group, the organ preservation rate was higher in the CRT-CNCT group (45% vs 33%), and more seriously, the tumor regrowth rate was unexpectedly high in the INCT-CRT group (40%). This seems to indicate that when considering the W&W strategy, CRT-CNCT may be the preferred treatment for LARC. But the reason for this phenomenon may be that compared to the INCT-CRT group, the CRT-CNCT group has a longer assessment interval (TI), which may allow more ncCR patients to transition to cCR status. We recommend that INCT-CRT should still be a viable treatment option in cases where there is no difference in survival outcomes between the two TNT regimens and high regrowth rates cannot be further clearly explained.

Unlike the OPRA trial, the CAO/ARO/AIO-12 study required TME after preoperative chemoradiotherapy. The results of this study exhibited that CNCT following CRT resulted in higher rates of pCR compared to induction chemotherapy followed by CRT and TME. Specifically, the 10-year cumulative incidence of distant metastasis was significantly lower for patients with complete regression (TRG 4), showing a rate of 10.5%, compared to 29.3% for intermediate regression (TRG 2 and 3) and 39.6% for poor regression (TRG 0 and 1). DFS was also notably higher at 89.5% for TRG 4, compared to 73.6% for intermediate and 63% for poor regression (3). The higher pCR rates in patients receiving CNCT can be attributed to the extended tumor exposure to systemic chemotherapy, which targets residual tumor cells weakened by the preceding radiotherapy, thus improving the complete regression outcome. This study provides some supporting evidence for the effectiveness of CNCT in preserving organ function, as observed in the OPRA trial.

### 2.3 Neoadjuvant immunotherapy

The therapeutic efficacy of immunotherapy has been widely acknowledged in advanced colorectal cancer patients with deficient DNA mismatch repair (dMMR) or high microsatellite instability (MSI-H). A recent cohort study observed a remarkable complete response rate of 90% among 20 LARC patients with dMMR/MSI-H after undergoing 7 cycles of neoadjuvant immunotherapy (53). Conversely, for patients with proficient DNA mismatch repair (pMMR) or microsatellite stability (MSS), several phase II trials have indicated that combining neoadjuvant radiotherapy with immunotherapy yields a higher rate of pCR (54–56).

In conclusion, compared to nCRT, TNT significantly improves clinical response and R0 resection rates, while exhibiting lower toxicity. Currently, TNT combined with TME has been proven to enhance long-term survival. Additionally, for patients with cCR following TNT treatment, W&W offers a promising alternative, potentially avoiding the risks of surgery and organ resection. Regarding the choice of TNT regimen, the CNCT scheme, which involves a longer interval from nCRT to surgical decision and more complete tumor regression, may be more suitable for patients who prioritize organ preservation. In contrast, for patients at high risk of tumor metastasis, the INCT regimen is a more appropriate option for early systemic control. Although replacing long-course radiotherapy with short-course radiotherapy in TNT appears to improve therapeutic outcomes, further large-scale studies are needed to confirm these findings. Regarding systemic chemotherapy in TNT, adding irinotecan to the traditional regimen may enhance tumor regression and survival, while incorporating immunotherapy can yield satisfactory tumor regression rates and improve oncologic prognosis.

## 3 Assessment of clinical complete response

### 3.1 Definition of cCR, near-cCR, pCR

pCR was defined as the absence of any residual tumor cells detected in the operative specimen, both at the primary tumor site and regional lymph nodes (ypT0N0Mx).

The diagnostic criteria for cCR currently involve digital anal examination (DRE), endoscopy, and pelvic high-resolution MRI. These methods are used to determine if certain conditions are met:

1. The original tumor area appears normal during DRE, and no palpable tumor mass is detected.
2. Under endoscopy, there are no signs of tumor or only a few residual erythematous ulcers or marks.
3. Pelvic high-resolution MRI shows substantial reduction in tumor size, with no observable residual tumor mass or only limited DWI signal indicating residual fibrosis. In some cases, residual intestinal wall thickening due to edema may be present, and there are no suspicious lymph nodes (57).

However, the current criteria for cCR still face challenges in terms of the accuracy of diagnosis. A previous study investigated expert opinions on non-surgical treatment after neoadjuvant therapy, and the results were shocking: 122 experts proposed over 70 different combinations of survey and imaging methods to define cCR (58). On one hand, the criteria are too lenient, allowing some patients who have not reached the “safe range” to be included in the W&W cohort. This can lead to local regrowth and distant metastasis. Previous studies have revealed that the proportion of cCR patients who actually achieve pCR after surgical resection is only 25% (59). For example, in a study involving 880 patients with cCR from the W&W database, the 2-year local regrowth rate was 25.2%, and the distant metastasis rate was 8% (60). Other studies have also indicated that the rate of local regrowth in patients achieving cCR can reach approximately 25% with a W&W strategy (61–64). On the other hand, the criteria might be too strict, resulting in many patients who could have been evaluated for complete clinical response undergoing RS instead. This approach carries a higher risk of complications and mortality rates. In a retrospective study, a cohort of 282 patients who underwent post-chemoradiotherapy or high-dose-rate brachytherapy (HDRBT) TME was included (65). Among these patients, 21.2% (60 patients) achieved a pCR, while only 3.2% of patients were classified as achieving a cCR after neoadjuvant therapy. This discrepancy can be attributed to the presence of residual mucosal abnormalities that prevent the attainment of cCR. Therefore, there is a need to strike a balance in the judgment of cCR criteria to avoid both under- and over-treatment of patients.

The concept of near complete clinical response (ncCR) was introduced to address the issue of defining tumor regression after nCRT. The MSKCC criteria, developed by a study led by MSKCC and involving 20 clinical centers, classified the degree of tumor regression into three categories: complete clinical response (cCR), near complete clinical response (ncCR), and incomplete clinical response (iCR) (66). This was the first attempt to classify tumor regression after nCRT. The China W&W Database Study Group (CW&WD) further refined the definition of ncCR. According to their definition, after neoadjuvant radiotherapy, physical examination and adjuvant assessments showed substantial tumor response in the form of scar tissue, but the tumor did not meet the diagnostic criteria for cCR (67). Hupkens et al. reported that among 49 patients initially identified as ncCR, the evaluation was extended up to 13-49 weeks (median: 23 weeks) after nCRT, and ultimately, 44 of these patients (90%) achieved cCR and proceeded with the W&W strategy (68). So this highlights the importance of choosing the optimal timing for cCR evaluation.

The optimal timing for evaluation is further clarified by guidelines. The ESMO recommends an interval of 6 to 8 weeks for the first evaluation, while the NCCN guidelines suggest an interval of 5 to 12 weeks (27, 28). A clinical trial conducted in Lyon, France, randomized 210 patients with cT2-3Nx resectable rectal cancer to neoadjuvant radiotherapy (13×3Gy) followed by surgery within either 2 weeks or 6-8 weeks. The results showed no significant difference in the pCR rate between the two groups. However, the group with the longer interval (6-8 weeks) had a higher incidence of ypT0 or ypT1 pathological stages (15% vs. 29%). This led to the adoption of 6-8 weeks as the optimal interval for evaluation (69).

The timing of the first evaluation also depends on the neoadjuvant treatment modality. For SCRT, surgical evaluation typically occurs 1 week after treatment completion. In contrast, for LCRT, evaluation generally occurs 6-8 weeks after treatment ends. However, LCRT has the disadvantage of longer treatment cycles and lower patient compliance. Despite this, LCRT provides a longer tumor regression period, resulting in a higher clinical remission rate than SCRT (70). TNT, which incorporates systemic adjuvant chemotherapy before RS, further complicates the timing. Due to the additional chemotherapy, TNT results in a longer preoperative course and further tumor cell destruction, complementing the tumor regression caused by nCRT. Consequently, the time interval between neoadjuvant therapy and surgery is extended. In the INCT group, the interval from treatment initiation to tumor response assessment and surgical decision was 25 to 36 weeks, while in the CNCT group, it ranged from 32 to 37 weeks (71, 72).

In the case of patients with ncCR, although there is still no clear “cutoff” time for assessment, reassessment can occur after a short interval from the initial determination to 12 weeks later. The time and method of evaluating cCR in some trials are shown in **Table 1**. However, the diagnosis of ncCR remains a topic of controversy, and future investigations focusing on defining its criteria could bridge the gap between pCR and cCR diagnoses.

**Table 1:** cCR evaluation methods of some studies.

Trail	Staging	Therapy(Therapy time)	cCR evaluation time(from start of therapy)	cCR evaluation project
STAR-TREC (NCT02945566)	cT1-T3bN0 ≤10cm AV	SCRT(5days)	First: 11-13 weeks Second:16-20weeks	MRI and endoscopy
		LCRT(5weeks)		
W&W3 (NCT04095299)	cT1-T3bN0 ≤10cm AV	CRT(4weeks)	Within 16 weeks	Unmention
		CRT+SIB(4weeks)		
OPERA (NCT02505750)	cT2-3bN0-1 ≤10cm AV	CRT(5weeks)	14weeks	MRI, DRE and endoscopy
		CRT+brachytherapy boost (11weeks)		
GRECCAR12 (NCT02514278)	cT2-3N0-1 ≤10cm AV	CRT(5weeks)	Approximately 24 weeks	DRE and MRI
		Induction chemotherapy + CRT (15-17weeks)		
ACO/ARO/AIO-18.1 (NCT04246684)	cT3C-T4N0/N+ ≤12 cm AV	SCRT + consolidation chemotherapy(5days+18weeks)	22-24weeks	Clinical investigation, endoscopy and MRI
		CRT+ consolidation chemotherapy(6weeks+12weeks)		
OPRA (NCT02008656)	cT3-T4N0/N+ ≤6 cm AV	Induction chemotherapy + CRT(21-24weeks)	25-36weeks	DRE, endoscopic examination, MRI, and CT
ENSEMBLE-1 (jRCT s051200113)	cT3-4 N0 M0 or Tany N+ M0 ≤12cm AV	SCRT+consolidation chemotherapy(5days+18weeks)	Approximately 20-28 weeks	MRI, colonoscopy and DRE
ACCORD 12/PRODIGE 2 (NCT00227747)	cT3-4 M0 ≤6cm AV	CRT (5weeks)	Approximately 6-8 weeks	DRE, ERUS and/or MRI
TEHRAN (NCT05920928)	cT3-4, N+ 5-15cm AV	SCRT(5days)	18weeks	MRI, colonoscopy and/or PET scan
		LCRT(5weeks)		
ENSEMBLE (NCT05646511)	cT3-4N0M0 or T1-4N1-2M0 ≤12cm AV	SCRT+consolidation chemotherapy(5days+18weeks)	Approximately 20-22 weeks	Unmention

AV, anal verge; CRT, chemoradiotherapy; cTNM, clinical TNM staging; DRE, digital rectal examination; SCRT, short-course radiotherapy; SIB, simultaneous integrated boost of radiotherapy; ERUS, endorectal ultrasound; LCRT, long course concurrent chemoradiation.

### 3.2 Imaging evaluation of cCR

To address the discrepancies between pCR and cCR, some clinicians have suggested the use of endoscopic biopsies due to the low rate of concordance between them (73, 74). However, a study conducted by Duldulao et al. demonstrated that residual cancer cells after nCRT were predominantly found in the muscularis propria, with only 13% of cancer cells in ypT stage 2-4 tumors located in the mucosal layer and 56% in the submucosal layer (75). Another study by R. O. Perez et al. revealed that biopsies performed after nCRT had an accuracy of only 21% in determining the absence of tumor cell remnants (76). The Clinical Practice Guidelines of the Chinese Watch and Wait also do not recommend routine endoscopic biopsies during nCRT follow-ups (67). Conversely, transanal multipoint whole-mount puncture biopsy (TMFP) has proven to be more clinically relevant. It demonstrated an accuracy of 94.4% for *in vivo* puncture and 83.3% for ex vivo puncture in determining pCR (*χ* ^2^ = 1.382, *P*=0.240) (77).

Conventional imaging techniques such as MRI and EUS are of more limited value in determining cCR after nCRT (78). However, recent advancements in imaging evaluation have the potential to address this limitation. Safatle-Ribeiro et al. conducted a study and found that Probe-Based Confocal Laser Endomicroscopy (pCLE) scores exhibited superior sensitivity, specificity, positive predictive value, negative predictive value, and accuracy in diagnosing persistent cCR as compared to MRI (66.7% vs. 66.7%, 93.5% vs. 48.4%, 80% vs. 66.7%, 88.9% vs. 78.9%, and 86% vs. 53.5%, respectively) (79). Furthermore, another study by Safatle-Ribeiro et al. demonstrated the diagnostic improvement in cCR using pCLE (80).

Another scoring system used in assessing neoadjuvant post-cCR is the magnetic resonance imaging tumor regression grade (mrTRG). It categorizes cCR determined by MRI after neoadjuvant therapy as mrTRG 1, while near cCR is classified as mrTRG. Studies have shown that mrTRG 1 at initial restaging predicts persistent cCR and the likelihood of organ preservation, with minimal chances of tumor regrowth within two years. Conversely, most patients with mrTRG 2 will have persistent tumors at initial restaging, necessitating surgery (81–86). However, a recent study by Sean J Judge et al. investigated the effect of the presence of residual mucin on MRI after neoadjuvant therapy on the evaluation of cCR and found that the presence of mucin after neoadjuvant therapy did not affect the W&W strategy for LARC patients who achieved an endoscopic clinical complete response (87).

The combination of different imaging modalities has shown promising results in improving the accuracy of response assessment in rectal cancer. The use of mrTRG along with the apparent diffusion coefficient (ADC), diffusion-weighted imaging (DWI), fluoro-D-glucose (FDG), and dynamic contrast-enhanced MRI (DCE-MRI) has been demonstrated to enhance the assessment of response in rectal cancer (82, 88–90).

Another imaging technique that holds potential for evaluating the effectiveness of nCRT is Positron Emission Tomography combined with Computed Tomography (PET-CT). In a prospective study of 99 patients with cT2-4N0-2M0 distal rectal cancer, PET-CT evaluations were performed at baseline, week 6, and week 12 after neoadjuvant therapy (91). The study found that the baseline primary tumor standardized uptake value (SUV) was a significant predictor of response, with a reduction of over 67% between baseline and 6 weeks, and a 76% reduction between baseline and 12 weeks in SUVmax, which correlated with complete remission (pCR or cCR; *P* = 0.02 and *P* < 0.001, respectively). Another study by Dalton A Dos Anjos et al. similarly demonstrated that PET-CT could be a valuable tool for predicting the response of distal rectal cancer to neoadjuvant radiotherapy (92).

Additionally, Ross K. McMahon et al. developed an important imaging-based neoadjuvant rectal (NAR) score that has demonstrated effectiveness in predicting preoperative overall survival (OS) and recurrence-free survival (RFS) (93). These findings contribute to a better understanding of the potential value of imaging in predicting treatment response and outcomes in patients with rectal cancer.

In summary, DRE is a convenient method that can detect minor abnormalities not identified by endoscopy or imaging. It is often used as a basic tool to assess tumor regression. However, it requires a high level of clinical experience from the examiner, which affects its stability and reproducibility. Endoscopy, as the cornerstone for evaluating cCR in tumors after nCRT, faces challenges due to the diversity of tumor regression patterns (94). From the perspective of advancing the W&W strategy, it is worthwhile to reconsider whether endoscopy should continue to be the primary method for assessing tumor regression. Compared to rectal examination and endoscopy, MRI offers a broader field of view (FOV) and can visualize the mesenteric fascia outside the intestinal lumen. MRI is also more objective than rectal endoluminal ultrasound, as it is less dependent on the examiner’s experience. This makes MRI a more reliable tool for assessing changes in tumor regression during nCRT. TMFP is safe, and feasible, and enhances the sensitivity and accuracy of determining pCR in rectal cancer after nCRT. It provides a solid pathological foundation for determining cCR and is the preferred approach when considering eligibility for the W&W strategy. The pCLE scoring system, based on epithelial and vascular features, improves the diagnosis of persistent cCR and is recommended for use during follow-up. On the other hand, 18F-FDG PET/CT, although valuable, is not yet sufficiently accurate. Its complexity, along with high diagnostic costs, limits its clinical applicability. Moreover, there is a lack of robust evidence supporting its role in evaluating tumor regression (95). The mrTRG scoring system, based on T2-weighted imaging (T2WI) sequences, has shown better efficacy in predicting pCR. When combined with DWI sequences, its accuracy improves further. However, challenges remain, such as issues with inter-observer agreement, diagnostic efficacy, and alignment with pathological findings (96). Imaging histology has also shown a promising role in assessing the efficacy of nCRT and is expected to realize clinical applications with the development of artificial intelligence technology (97, 98).

### 3.3 Evaluation of blood markers for cCR

In addition to utilizing imaging evidence, further assessment of the risk of rectal cancer recurrence can be strengthened by analyzing blood biomarker. Researchers have demonstrated that circulating tumor DNA (ctDNA) serves as a reliable predictor for recurrence risk following TME (99–102). Wang et al. developed a prediction model that combines ctDNA and mrTRG to effectively anticipate the response to nCRT and provide valuable guidance to potentially avoid unnecessary surgery (103).

A liquid biopsy indicator, known as circulating tumor cells (CTC), has also shown promise in predicting the prognosis of patients undergoing surgery for advanced rectal cancer. A recent study exhibited that patients who experienced a reduction of more than 1 in their CTC count following radiotherapy had higher rates of pCR and sustained cCR (HR, 4.00; 95% CI, 1.09-14.71, *P* = 0.037) (104). Several other studies have corroborated that dynamic testing of CTC can enhance risk assessment after neoadjuvant therapy for LARC (105, 106).

In a multicenter cohort study conducted in Ireland, 422 patients from three specialist centers for rectal cancer were included to assess whether inflammatory markers after nCRT can assist MRI and endoscopy in identifying cCR in rectal cancer (107). The study demonstrated that combining MRI and endoscopic cCR with a neutrophil-to-lymphocyte ratio (NLR) of less than 5 reflected significantly higher odds of achieving ypCR (OR 6.503; 95% CI 1.594-11.652; *P* < 0.001). Additionally, other blood markers such as T-cell factor 4 (TCF4), programmed cell death 4 (PDCD4), and circulating lymphocytes have all shown predictive potential for favorable tumor response and prognosis in patients with LARC undergoing nCRT (108–110).

In conclusion, ctDNA shows great potential in predicting the risk of cCR recurrence after neoadjuvant therapy. However, its clinical use is hindered by the lack of standardized analysis protocols and variations in assay performance across different studies, which means further standardization is needed before it can be widely implemented (111). Similarly, CTCs are a safe and minimally invasive alternative to radiological scans and colonoscopies, offering real-time monitoring of cancer efficacy and recurrence. Despite these advantages, technical limitations still impede its broader clinical application (112). Tumor tissue- or serum-based proteomics can generate large amounts of valuable data for predicting response to nCRT in patients with LARC. However, most of these studies lack robust validation, making it difficult to establish convincing correlations that could be translated into clinical practice. Additionally, proteomics is influenced by factors such as tumor heterogeneity, sample source, sample processing, and mass spectrometry instrumentation, which can complicate its clinical implementation.

## 4 Management of patients with cCR after neoadjuvant therapy

### 4.1 Total mesorectal excision

The recommendations provided by both the NCCN and ESMO guidelines emphasize the use of TME as the primary treatment for patients with LARC who have undergone nCRT or TNT (27, 28). In a Chinese clinical trial involving 238 patients with stage II-III LARC who achieved cCR after nCRT, 59 patients underwent the W&W approach, while 179 patients received TME after 6-12 weeks (113). The study aimed to compare the long-term efficacy of W&W and TME in LARC patients with cCR after nCRT. The results showed that the 3-year local recurrence rate (LRR) in the W&W group was 12.9% (7 cases relapsed within 2 years), significantly higher than the 0.6% LRR observed in the TME group (P=0.003). For patients with a tumor distance from the anal verge of ≤5 cm, the sphincter preservation rate (SPR) in the W&W group was 88.0%, which was significantly higher than the 54.4% in the TME group (P<0.001). Another retrospective meta-analysis showed that for patients with fatal cancer with clinical complete response after nCRT, a higher risk of disease recurrence was observed in the non-operative management (NOM) group compared to the TME group (RR = 1.69, 95% CI 1.08, 2.64) and patients in the NOM group were more likely to experience local recurrence (RR = 5.37, 95% CI 2.56, 11.27). But patients in the TME group were more likely to have a permanent colostomy (RR = 0.15, 95% CI 0.08, 0.29) (114). While TME has shown efficacy in controlling distant metastasis and local recurrence in patients with LARC, its postoperative mortality rates have been less than optimal, potentially due to intraoperative tumor rupture (115).

In addition, TME can lead to varying degrees of intestinal dysfunction, including frequent defecation, urgency, incomplete evacuation, stool or gas incontinence, and other related symptoms. These symptoms are collectively called low anterior resection syndrome (LARS). A meta-analysis of the incidence of LARS after sphincter-preserving surgery for LARC found that the pooled incidence of LARS following TME was 44% (95% CI 40% to 48%) (116). Page et al. reported that diarrhea is one of the most common symptoms of LARS, with more than half of patients experiencing liquid fecal incontinence, which significantly impacts their quality of life (117). Van Heinsbergen et al. further noted that patients with severe LARS experienced a significant decline in nearly all general quality of life domains compared to patients with no or mild LARS (118). Anastomotic fistula, the most severe complication following intestinal surgery, can lead to sepsis and peritonitis, increasing postoperative mortality by 6% to 22% (119). Recently, a new approach called transanal TME (taTME) has gained global attention for the treatment of middle and low rectal cancer. This technique uses a “bottom-up” approach to improve the limited field of vision in traditional TME, offering a higher recognition rate of local nerve structures during surgery. This innovation helps reduce damage to vascular and nerve bundles and is a promising direction for minimizing the occurrence of LARS (120).

### 4.2 Local excision

TME surgery, while effective for rectal cancer treatment, may not always address patient concerns regarding body image and health-related quality of life (HRQL). As a result, the possibility of opting for local excision has been raised as an alternative. Local excision (LE) techniques include traditional transanal endoscopic microsurgery (TEM) or transanal minimally invasive surgery (TAMIS) (121). However, there is ongoing debate regarding the appropriateness of local excision for patients who achieve clinical remission or complete clinical response after neoadjuvant therapy.

A study from the United Kingdom evaluated rectal cancer patients with clinical stage T1 or T2N0M0 who received short-course radiation therapy followed by local excision (122). Out of the 62 patients who underwent local excision, 7 patients (11%) experienced recurrent disease postoperatively, while postoperative fistula formation or the need for a stoma was rare. Another phase II clinical trial conducted at multiple centers expanded the patient staging to T2-T3. In this trial, LE was performed after a favorable clinical response following preoperative radiotherapy, with additional TME performed for patients with postoperative staging beyond ypT0-1 (123). The 3-year OS, disease-free survival, and localized disease-free survival rates were reported as 91.5%, 91.0%, and 96.9% respectively.

The CARTS study conducted in the Netherlands modified the inclusion criteria to cT1~3N0 and demonstrated that local excision was feasible in around two-thirds of patients with this baseline staging after nCRT. The study reported 5-year RFS and OS rates of 81.6% and 82.8% respectively. Notably, patients who underwent local excision experienced significant improvements in quality of life (124).

The GRECCAR 2 study, a multicenter trial comparing LE to TME, was the first of its kind (125). The study comprised 145 patients diagnosed with clinical stages I to III who responded well to neoadjuvant therapy. The primary endpoints assessed after 2 years of surgery included death, recurrence, morbidity, and side effects. However, the three-year and five-year follow-up results of the study failed to demonstrate the superiority of LE over TME. It is worth noting that some patients in the localized resection group underwent additional TME procedures, which may have contributed to diminishing the overall advantage of the localized resection group compared to the TME group.

In a randomized trial conducted by Teste, patients with clinical stage T2 or T3 and N0-1 were enrolled to compare the effectiveness of LE versus TME in staged tumors after neoadjuvant radiotherapy for low-grade rectal cancer (126). The study observed that, owing to the resolution of early postoperative complications and the body’s gradual adaptation to surgical changes, the rate of surgical complications following neoadjuvant radiotherapy in the LE group was significantly lower compared to the TME group. Specifically, at one-month post-surgery, the complication rate in the LE group was approximately half that of the TME group, and this difference became even more pronounced at two years, with the LE group exhibiting a tenfold reduction in complication rates.

Furthermore, the latest ReSARCh study from Italy focused on 160 cases with clinical staging cT1-4N0-2b and aimed to explore the efficacy of LE and wait-and-watch strategies in achieving complete clinical response (mCR or cCR) after radiotherapy (127). In this study, the incidence of serious postoperative complications after LE was found to be as low as 3.1%, indicating a favorable safety profile.

Several studies have underscored the significance of pre-treatment baseline in rectal cancer. For instance, a study examined the response to surgery in 27 T0/T1 tumors and found that 13 achieved a complete pCR. However, among the 29 tumors classified as T2, only 5 achieved pCR (128). In another study conducted by Perez et al., a prospective analysis of 27 patients who underwent local excision pathology after neoadjuvant radiotherapy revealed a high LRR of up to 15% over a median follow-up of 15 months. Further analysis indicated a higher proportion of patients with baseline staging of cT3 and cN1 (55.6% and 18.5%, respectively), which correlated with the increased risk of local recurrence (129).

### 4.3 Watch-and-wait strategy

Non-operative treatment of rectal cancer is appealing due to the high rates of complications, mortality, and functional consequences associated with TME procedures. However, the W&W strategy does not completely eliminate the need for surgery as some patients may experience local tumor regrowth or distant metastases, necessitating additional surgical intervention (130). The occurrence of local regrowth and distant metastases is strongly influenced by the baseline levels before treatment. A recent meta-analysis involving 1,254 patients from 14 moderate to high-quality studies compared two treatment approaches: W&W and RS. Of the participants, 513 patients received the W&W strategy, while 741 underwent RS. The results revealed that the W&W group had a significantly higher incidence of local recurrence compared to the RS group (odds ratio [OR] = 11.09, 95% confidence interval [CI] = 5.30–23.20, P = 0.000). However, the W&W group had a significantly lower incidence of permanent colostomy (OR = 0.12, 95% CI = 0.05–0.29, P = 0.000) (131). RS requires the creation of a permanent sigmoidostomy, as it cannot preserve the normal function of the anus. This procedure replaces the original defecation function of the perineal anus. In contrast, under the W&W strategy, only patients who experience local regrowth must undergo remedial RS. In another meta-analysis comparing patients who achieved cCR after neoadjuvant therapy, the W&W strategy was compared to both LE and RS. The results indicated that the W&W approach did not significantly increase the risk of local recurrence compared to local resection (relative risk [RR] = 1.12, 95% CI = 0.73–1.72, P = 0.593). However, compared to RS, the W&W strategy was associated with a greater risk of local recurrence (RR = 2.09, 95% CI = 1.44–3.03, P < 0.001). On the other hand, the W&W group showed a significantly lower stoma rate compared to the surgery group (RR = 0.35, 95% CI = 0.20–0.61, P < 0.001) (132). While the W&W strategy did not increase the risk of local recurrence relative to local excision, it may have a higher risk of local recurrence compared to RS; however, the rate of stoma formation was significantly better in the W&W group compared to the surgical group. These studies underscore the superiority of non-surgical treatment over RS in terms of improving quality of life. Nevertheless, it remains unclear whether this evidence sufficiently supports the adoption of a W&W strategy. Some scholars argue that comparing TME and W&W strategies introduces bias since patients with residual tumors requiring TME after neoadjuvant therapy would never be considered for a W&W strategy (130). This raises the importance of pre-treatment baseline assessment to determine the suitability of surgery or the W&W approach for each patient.

In a prospective study involving 71 patients, the W&W approach was used with a median follow-up of 24 months. The study observed a cCR rate of 39.0% for patients with cT1-2N0 tumors, 16.8% for patients with cT3 tumors exhibiting unthreatened rectal mesenteric fascia (MRF-), and 5.4% for patients with cT4 or MRF+ tumors (133). Notably, circumscribed carcinomas or tumors ≥7 cm in length demonstrated a lower cCR rate of only 2.7%. Previous studies have suggested that tumor size is related to chemoradiotherapy sensitivity, and tumor T staging is also related to tumor size. Therefore, higher pre-treatment T staging is associated with highly invasive tumor behavior, lower sensitivity to nCRT, and a lower rate of stage decline (134, 135).

Contrastingly, in a retrospective study, patients with cT2 and cT3-4 stages exhibited similar cCR rates. However, the rate of early localized regrowth was significantly higher in patients with cT3-4 stages compared to those with cT2 stages. Additionally, the overall prognosis for patients with localized regrowth who underwent salvage surgery remained inferior to that of patients without localized regrowth (136). These findings emphasize the superior prognosis of the W&W strategy for rectal cancers with early baseline staging. Nevertheless, it is important to note that previous studies evaluating non-surgical treatments included cases with widely varying baseline levels and lacked subgroup analyses with relevant baselines (137–139).

The W&W strategy is challenging because of the potential for local regrowth and distant metastasis. Studies have shown that approximately 25% to 30% of patients who achieve cCR by nonsurgical treatment eventually develop local regrowth (50, 54, 120, 121). Additionally, the OPRA trial suggests that patients with initial cCR or ncCR following W&W and subsequent local regrowth may face an increased risk of distant metastasis after receiving TNT. A multicenter study from the International Watch and Wait Database (IWWD) across 15 countries, which included 880 patients with cCR to rectal cancer, investigated key outcomes such as local recurrence, distant metastasis, 5-year OS, and 5-year disease-specific survival (DSS). The results showed that the 2-year cumulative incidence of local recurrence and distant metastasis were 25.2% and 8.0%, respectively, with 95% of local recurrences occurring in the intestinal wall. Furthermore, the 5-year OS rate was 85.0%, and the DSS rate was 94.0% (60). Notably, the incidence of local recurrence appeared to level off after 3 years, with few patients developing regrowth beyond 5 years post-W&W decision. A meta-analysis of survival outcomes for the W&W strategy in patients with cCR after nCRT found no significant difference in distant metastasis rates between the W&W approach and RS (140). Preliminary studies indicate that the baseline T stage is the most significant risk factor for local regrowth and distant metastasis. However, HAN et al. (141) found that patients with a tumor located ≤5 cm from the anal verge had a significantly higher incidence of lung metastasis (65%) compared to those with tumors 10-15 cm from the anal verge (35%). AKIYOSHI (142) demonstrated that patients with positive lymph node metastasis had a significantly higher LRR (16.3%) compared to those with negative lymph node metastasis (5.5%). Thus, lymph node metastasis and tumor location influence regrowth and recurrence. A recently reported multicenter collaborative study investigated the role of local regrowth in promoting the spread of distant metastases (143). Of 793 patients, 85 (10.7%) developed metachronous distant metastasis, with local regrowth leading to a 5-fold increased risk of distant metastasis. Therefore, local regrowth and distant metastasis also have a close relationship.

For patients experiencing local tumor regrowth during the W&W period, remedial TME surgery is the standard treatment. However, patients who achieve near-cCR and face difficulties in preserving anal function may opt for local excision and should be reintroduced into the W&W follow-up program post-surgery. In cases where patients maintain cCR locally but develop distant metastases, priority should be given to treating the distant metastases while continuing to observe the primary lesion. A meta-analysis comparing W&W and RS for salvage surgery outcomes in rectal cancer patients with cCR after neoadjuvant radiotherapy found no significant difference in distant metastasis rates. Although local recurrence rates were higher in the W&W group, similar long-term outcomes were observed following salvage surgery (140). A retrospective study by Daniela Rega et al. confirmed this finding, showing no difference in local recurrence rates or oncologic outcomes between primary and advanced TME surgeries (144). Additionally, patient-reported quality of life was preserved following salvage resection for locally recurrent disease, whereas it deteriorated rapidly in patients with local recurrence who did not undergo salvage surgery.

### 4.4 Measures to monitor recurrence

The risk of local regrowth is most common within three years after achieving a cCR, while the risk of distant metastases is relatively low in patients who achieve cCR through treatment, the long-term follow-up results of 880 cCR patients in the IWWD showed that 25.2% of patients experienced local regrowth and 8.0% of patients experienced distant metastasis (60, 145–147). Therefore, in the case of LARC (stage II, III), a monitoring schedule is followed: evaluations are conducted every three months during the first three years, then with reduced frequency, every six months, up to five years. Even after five years of being free from the disease, lifelong surveillance is recommended, as there remains a possibility of regrowth, albeit very low (148, 149). The main objective of surveillance is to detect metastatic recurrences that can still be treated effectively with radical-oriented therapy. However, despite considering the economic implications, there is currently no evidence to suggest the best monitoring strategy. The consensus of follow-up strategies reached so far is shown in **Table 2**.

**Table 2:** Follow-up methods and intervals for organ preservation strategies.

Timeline(year)	1				2				3				4		5+
Month	3	6	9	12	15	18	21	24	27	30	33	36	42	48	
CEA	√	√	√	√	√	√	√	√	√	√	√	√	√	√	Same as the 4th year
DRE	√	√	√	√	√	√	√	√		√		√	√	√	
Rectoscopy	√	√	√	√	√	√	√	√		√		√	√	√	
Rectum MRI	√	√	√	√	√	√	√	√		√		√	√	√	
Chest and/or abdominal CT		√		√				√				√		√	
Biopsy	When rectoscopy is abnormal														

DRE, digital rectal examination; CEA, Serum carcinoembryonic antigen.

√ denotes the items that should be monitored at the corresponding follow-up time point.

## 5 Discussion and prospects

Before discussing the management of cCR patients, the first thing to consider is the evaluation time of cCR. The evaluation time point of cCR is one of the biggest problems at present because the most appropriate time point for evaluating cCR is affected by many factors, such as tumor size, histology, different chemoradiotherapy strategies, time after completion of chemoradiotherapy, and different evaluation methods. A meta-analysis compared the classical interval (less than 8 weeks) and at least 8-week interval pCR rates of LARC patients from nCRT to TME, with a minimum 8-week interval was associated with increased odds of pCR (OR= 1.41, 95% CI[1.30, 1.52]; *P*<0.001) (150). Any strategy that increases pCR rates will increase the number of patients deemed to have a clinical complete response (cCR), however, the reason for the pCR rate caused by extending the interval is still unclear. In addition, the introduction of whole course neoadjuvant therapy with more complex chemoradiotherapy regimens further increases the uncertainty of the evaluation time point. The author suggests selecting appropriate evaluation time points based on different treatment plans. For early-stage tumors and patients treated with LCRT (5 weeks) or SCRT (5 days), the first evaluation was conducted at 11-14 weeks, and the second evaluation was conducted at 16-20 weeks, which has a more accurate judgment for ncCR patients; For patients treated with TNT (including induction chemotherapy and CNCT), cCR evaluation is recommended at 20-24 weeks. For the diagnosis of cCR and ncCR, besides DRE, MRI, and rectoscopy, PET-CT, biological, and blood markers can also be considered for the evaluation of cCR, but they are not mandatory.

The ESMO recommends the W&W strategy in two specific situations: (1) for patients with low rectal cancer at initial staging cT1-2N0, where RS for anal preservation is difficult, but there is a strong desire to preserve the anus, and (2) for patients with low and intermediate rectal cancer at initial staging cT3-4N+, who achieve cCR after neoadjuvant therapy. Despite its advantages, the W&W strategy has been questioned due to concerns about local regrowth and distant metastasis. However, the NCCN suggests that based on long-term follow-up studies with large sample sizes of patients who have achieved cCR in rectal cancer, and with the support of a multidisciplinary team experienced in diagnosis and treatment, a clinical study of the W&W approach can be considered. For patients with LARC who achieve cCR after receiving neoadjuvant therapy, or those who remain ncCR after two assessments and desire organ preservation, it is recommended to consider the W&W strategy. This approach can help avoid unnecessary TME surgery, improve quality of life, and maintain the curative effect, provided that patients are fully informed, have good compliance, and can be closely monitored through regular follow-up assessments (66).

**Figure 2:**
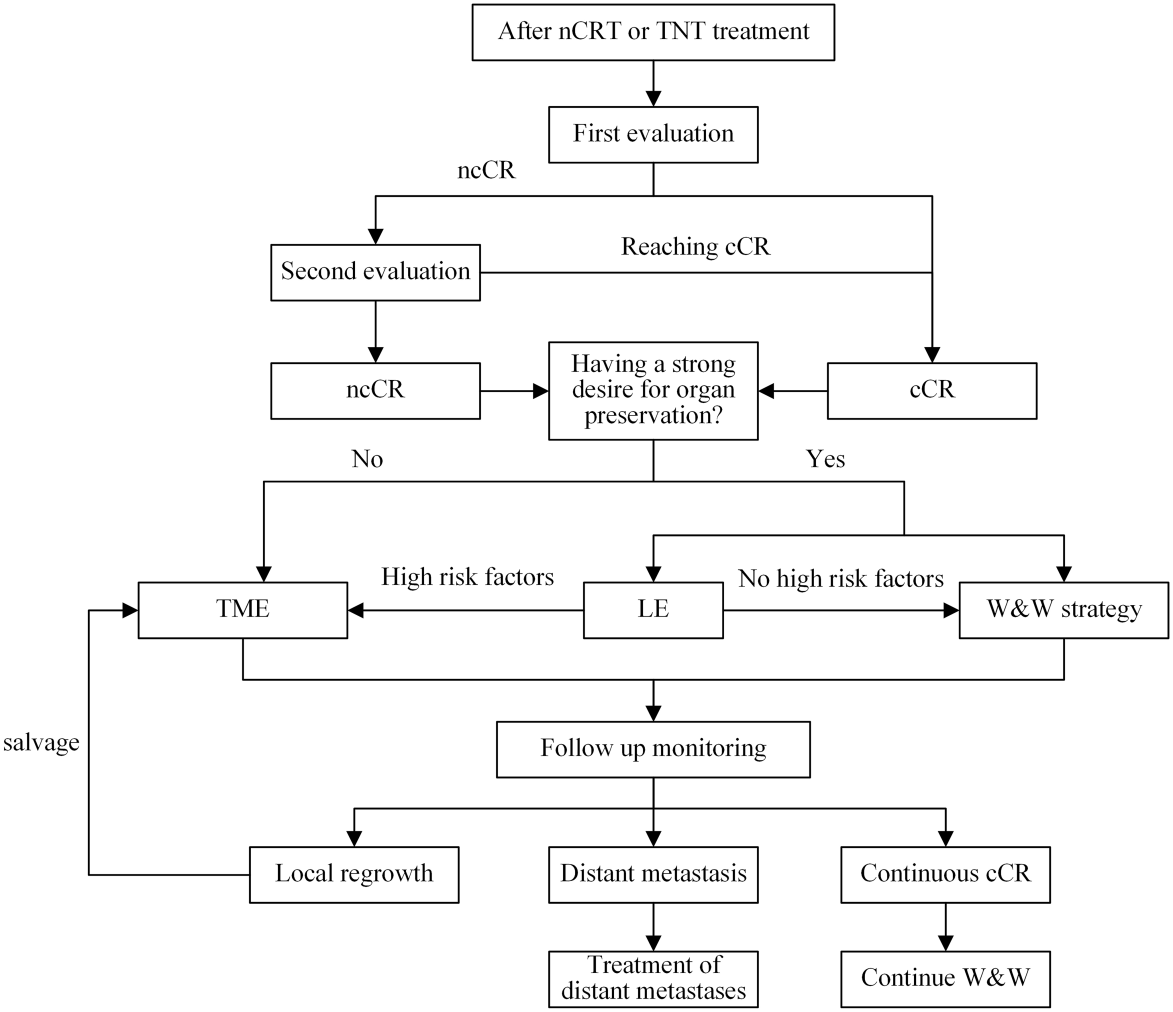
Management flow chart. nCRT, nCRT; TNT, total neoadjuvant therapy; ncCR, near-complete response; cCR, clinical complete response; TME, total mesorectal resection; LE, Local excision; W&W, watch and wait.

However, Le or W&W strategies are not recommended for patients with the following high-risk conditions: (1) mesorectal fascia+ (MRF+); (2) extramural venousinvasion+ (EMVI+), tumor invasion of the branches of the superior rectal vessels on imaging; (3) T3c~d/T4; (4) signet ring cell carcinoma(SRCC) or mucinous adenocarcinoma(MAC); (5) circular cancers or with a length ≥7 cm (133, 151). ncCR Patients with baseline <ycT2N0 may consider LE to preserve organ function and be reintegrated into W&W strategy follow-up after surgery, Moreover, in the case of poorly-differentiated adenocarcinoma, grade 2/3 tumor budding or lymphovascular involvement, TME is required due to the risk of associated lymph node invasion (28, 152).W&W’s follow-up strategies refer to international consensus recommendations. If distant metastasis occurs during the follow-up monitoring period, priority treatment should be given according to the treatment recommendations of guidelines (27, 28). If the primary lesion can maintain cCR, continue to observe. For patients with local tumor regeneration during the follow-up monitoring period, salvage TME is the main treatment recommended by the standard guidelines (153, 154). Salvage LE is not recommended because local excision of the scar significantly increases the complication rate of subsequent TME surgery (125). The management flow chart is shown in **Figure 2**.

## References

[1] Davidson KW Barry MJ Mangione CM Cabana M Caughey AB Davis EM. Screening for colorectal cancer: US preventive services task force recommendation statement. Jama. (2021) 325:1965–77. doi: 10.1001/jama.2021.6238 34003218

[2] Moore JS Aulet TH. Colorectal cancer screening. Surg Clin North Am. (2017) 97:487–502. doi: 10.1016/j.suc.2017.01.001 28501242

[3] Fokas E Liersch T Fietkau R Hohenberger W Beissbarth T Hess C. Tumor regression grading after preoperative chemoradiotherapy for locally advanced rectal carcinoma revisited: updated results of the CAO/ARO/AIO-94 trial. J Clin Oncol. (2014) 32:1554–62. doi: 10.1200/jco.2013.54.3769 24752056

[4] Li Y Wang J Ma X Tan L Yan Y Xue C. A review of neoadjuvant chemoradiotherapy for locally advanced rectal cancer. Int J Biol Sci. (2016) 12:1022–31. doi: 10.7150/ijbs.15438 PMC4971740 27489505

[5] Sauer R Becker H Hohenberger W Rödel C Wittekind C Fietkau R. Preoperative versus postoperative chemoradiotherapy for rectal cancer. N Engl J Med. (2004) 351:1731–40. doi: 10.1056/NEJMoa040694 15496622

[6] Soudy H. Ongoing evolution of preoperative chemoradiotherapy for rectal cancer. ANZ J Surg. (2018) 88:8–9. doi: 10.1111/ans.14223 29392907

[7] Boublikova L Novakova A Simsa J Lohynska R. Total neoadjuvant therapy in rectal cancer: the evidence and expectations. Crit Rev Oncol Hematol. (2023) 192:104196. doi: 10.1016/j.critrevonc.2023.104196 37926376

[8] Cercek A Roxburgh CSD Strombom P Smith JJ Temple LKF Nash GM. Adoption of total neoadjuvant therapy for locally advanced rectal cancer. JAMA Oncol. (2018) 4:e180071. doi: 10.1001/jamaoncol.2018.0071 29566109 PMC5885165

[9] Goffredo P Quezada-Diaz FF Garcia-Aguilar J Smith JJ. Non-operative management of patients with rectal cancer: lessons learnt from the OPRA trial. Cancers (Basel). (2022) 14. doi: 10.3390/cancers14133204 PMC9264788 35804975

[10] Liu S Jiang T Xiao L Yang S Liu Q Gao Y. Total neoadjuvant therapy (TNT) versus standard neoadjuvant chemoradiotherapy for locally advanced rectal cancer: A systematic review and meta-analysis. Oncologist. (2021) 26:e1555–e66. doi: 10.1002/onco.13824 PMC8417863 33987952

[11] Burbach JP den Harder AM Intven M van Vulpen M Verkooijen HM Reerink O. Impact of radiotherapy boost on pathological complete response in patients with locally advanced rectal cancer: a systematic review and meta-analysis. Radiother Oncol. (2014) 113:1–9. doi: 10.1016/j.radonc.2014.08.035 25281582

[12] Habr-Gama A Sabbaga J Gama-Rodrigues J São Julião GP Proscurshim I Bailão Aguilar P. Watch and wait approach following extended neoadjuvant chemoradiation for distal rectal cancer: are we getting closer to anal cancer management? Dis Colon Rectum. (2013) 56:1109–17. doi: 10.1097/DCR.0b013e3182a25c4e 24022527

[13] Habr-Gama A São Julião GP Perez RO. Nonoperative management of rectal cancer: identifying the ideal patients. Hematol Oncol Clin North Am. (2015) 29:135–51. doi: 10.1016/j.hoc.2014.09.004 25475576

[14] Dijkstra EA Mul VEM Hemmer PHJ Havenga K Hospers GAP Muijs CT. Re-irradiation in patients with recurrent rectal cancer is safe and feasible. Ann Surg Oncol. (2021) 28:5194–204. doi: 10.1245/s10434-021-10070-6 PMC8349344 34023946

[15] Glynne-Jones R Hughes R. Critical appraisal of the ‘wait and see’ approach in rectal cancer for clinical complete responders after chemoradiation. Br J Surg. (2012) 99:897–909. doi: 10.1002/bjs.8732 22539154

[16] Xiao WW Li M Guo ZW Zhang R Xi SY Zhang XG. A genotype signature for predicting pathologic complete response in locally advanced rectal cancer. Int J Radiat Oncol Biol Phys. (2021) 110:482–91. doi: 10.1016/j.ijrobp.2021.01.005 33434612

[17] On J Aly EH. Watch and wait’ in rectal cancer: summary of the current evidence. Int J Colorectal Dis. (2018) 33:1159–68. doi: 10.1007/s00384-018-3116-5 29978363

[18] Asoglu O Bulut A Aliyev V Piozzi GN Guven K Bakır B. Chemoradiation and consolidation chemotherapy for rectal cancer provides a high rate of organ preservation with a very good long-term oncological outcome: a single-center cohort series. World J Surg Oncol. (2022) 20:358. doi: 10.1186/s12957-022-02816-7 36352416 PMC9646475

[19] Beard BW Rettig RL Ryoo JJ Parker RA McLemore EC Attaluri V. Watch-and-wait compared to operation for patients with complete response to neoadjuvant therapy for rectal cancer. J Am Coll Surg. (2020) 231:681–92. doi: 10.1016/j.jamcollsurg.2020.08.775 33121903

[20] Han Z Li M Chen J Ji D Zhan T Peng Y. Surgery may not benefit patients with locally advanced rectal cancer who achieved clinical complete response following neoadjuvant chemoradiotherapy. Asian J Surg. (2022) 45:97–104. doi: 10.1016/j.asjsur.2021.03.025 33888366

[21] Cerdán-Santacruz C Vailati BB São Julião GP Habr-Gama A Perez RO. Local tumor regrowth after clinical complete response following neoadjuvant therapy for rectal cancer: what happens when organ preservation falls short. Techniques Coloproctol. (2023) 27:1–9. doi: 10.1007/s10151-022-02654-5 35986804

[22] Akagi T Inomata M Fujishima H Fukuda M Konishi T Tsukamoto S. Preoperative chemoradiotherapy versus surgery alone for advanced low rectal cancer: a large multicenter cohort study in Japan. Surg Today. (2020) 50:1507–14. doi: 10.1007/s00595-020-02034-2 32524272

[23] Hong YS Kim SY Lee JS Nam BH Kim KP Kim JE. Oxaliplatin-based adjuvant chemotherapy for rectal cancer after preoperative chemoradiotherapy (ADORE): long-term results of a randomized controlled trial. J Clin Oncol. (2019) 37:3111–23. doi: 10.1200/jco.19.00016 31593484

[24] Åsli LM Johannesen TB Myklebust T Møller B Eriksen MT Guren MG. Preoperative chemoradiotherapy for rectal cancer and impact on outcomes - A population-based study. Radiother Oncol. (2017) 123:446–53. doi: 10.1016/j.radonc.2017.04.012 28483302

[25] Kobayashi H Sugihara K. Surgical management and chemoradiotherapy of T1 rectal cancer. Dig Endosc. (2013) 25 Suppl 2:11–5. doi: 10.1111/den.12068 23617642

[26] Li A Huang T Zheng R Chi P Li Z Wang X. Preoperative chemoradiotherapy with capecitabine and triweekly oxaliplatin versus capecitabine monotherapy for locally advanced rectal cancer: a propensity-score matched study. BMC Cancer. (2022) 22:789. doi: 10.1186/s12885-022-09855-z 35850711 PMC9295262

[27] Glynne-Jones R Wyrwicz L Tiret E Brown G Rödel C Cervantes A. Rectal cancer: ESMO Clinical Practice Guidelines for diagnosis, treatment and follow-up. Ann Oncol. (2018) 29:iv263. doi: 10.1093/annonc/mdy161 29741565

[28] Benson AB Venook AP Al-Hawary MM Azad N Chen YJ Ciombor KK. Rectal cancer, version 2.2022, NCCN clinical practice guidelines in oncology. J Natl Compr Canc Netw. (2022) 20:1139–67. doi: 10.6004/jnccn.2022.0051 36240850

[29] Kim DY Jung KH Kim TH Kim DW Chang HJ Jeong JY. Comparison of 5-fluorouracil/leucovorin and capecitabine in preoperative chemoradiotherapy for locally advanced rectal cancer. Int J Radiat Oncol Biol Phys. (2007) 67:378–84. doi: 10.1016/j.ijrobp.2006.08.063 17097835

[30] Rödel C Graeven U Fietkau R Hohenberger W Hothorn T Arnold D. Oxaliplatin added to fluorouracil-based preoperative chemoradiotherapy and postoperative chemotherapy of locally advanced rectal cancer (the German CAO/ARO/AIO-04 study): final results of the multicentre, open-label, randomised, phase 3 trial. Lancet Oncol. (2015) 16:979–89. doi: 10.1016/s1470-2045(15)00159-x 26189067

[31] Zhu J Liu A Sun X Liu L Zhu Y Zhang T. Multicenter, randomized, phase III trial of neoadjuvant chemoradiation with capecitabine and irinotecan guided by UGT1A1 status in patients with locally advanced rectal cancer. J Clin Oncol. (2020) 38:4231–9. doi: 10.1200/jco.20.01932 PMC7768334 33119477

[32] Deng Y Chi P Lan P Wang L Chen W Cui L. Neoadjuvant modified FOLFOX6 with or without radiation versus fluorouracil plus radiation for locally advanced rectal cancer: final results of the chinese FOWARC trial. J Clin Oncol. (2019) 37:3223–33. doi: 10.1200/jco.18.02309 PMC6881102 31557064

[33] Wo JY Anker CJ Ashman JB Bhadkamkar NA Bradfield L Chang DT. Radiation therapy for rectal cancer: executive summary of an ASTRO clinical practice guideline. Pract Radiat Oncol. (2021) 11:13–25. doi: 10.1016/j.prro.2020.08.004 33097436

[34] Shang J Kong W Wang Y-y Ding Z Yan G Zhe H. VMAT planning study in rectal cancer patients. Radiat Oncol. (2014) 9:219. doi: 10.1186/s13014-014-0219-1 25319073 PMC4205282

[35] Lin Z Cai M Zhang P Li G Liu T Li X. Phase II, single-arm trial of preoperative short-course radiotherapy followed by chemotherapy and camrelizumab in locally advanced rectal cancer. J Immunother Cancer. (2021) 9. doi: 10.1136/jitc-2021-003554 PMC8562535 34725214

[36] Jimenez-Fonseca P Salazar R Valenti V Msaouel P Carmona-Bayonas A. Is short-course radiotherapy and total neoadjuvant therapy the new standard of care in locally advanced rectal cancer? A sensitivity analysis of the RAPIDO clinical trial. Ann Oncol. (2022) 33:786–93. doi: 10.1016/j.annonc.2022.04.010 35462008

[37] Mullen TD Kim EY Apisarnthanarax S. Short-course radiation therapy versus long-course chemoradiation in the neoadjuvant treatment of locally advanced rectal cancer: new insights from randomized trials. Curr Colorectal Cancer Rep. (2017) 13:165–74. doi: 10.1007/s11888-017-0359-4

[38] Wu H Fan C Fang C Huang L Li Y Zhou Z. Preoperative short-course radiotherapy followed by consolidation chemotherapy for treatment with locally advanced rectal cancer: a meta-analysis. Radiat Oncol. (2022) 17:14. doi: 10.1186/s13014-021-01974-4 35073940 PMC8785003

[39] Bregni G Akin Telli T Camera S Deleporte A Moretti L Bali AM. Adjuvant chemotherapy for rectal cancer: Current evidence and recommendations for clinical practice. Cancer Treat Rev. (2020) 83:101948. doi: 10.1016/j.ctrv.2019.101948 31955069

[40] Boustani J Caubet M Bosset JF. Adjuvant chemotherapy in rectal cancer after chemoradiotherapy. Clin Oncol (R Coll Radiol). (2016) 28:140–5. doi: 10.1016/j.clon.2015.11.004 26698026

[41] Watanabe T. Chemoradiotherapy and adjuvant chemotherapy for rectal cancer. Int J Clin Oncol. (2008) 13:488–97. doi: 10.1007/s10147-008-0849-0 19093175

[42] Roselló S Papaccio F Roda D Tarazona N Cervantes A. The role of chemotherapy in localized and locally advanced rectal cancer: A systematic revision. Cancer Treat Rev. (2018) 63:156–71. doi: 10.1016/j.ctrv.2018.01.001 29407455

[43] Petersen SH Harling H Kirkeby LT Wille-Jørgensen P Mocellin S. Postoperative adjuvant chemotherapy in rectal cancer operated for cure. Cochrane Database Syst Rev. (2012) 2012:Cd004078. doi: 10.1002/14651858.CD004078.pub2 22419291 PMC6599875

[44] Ooi BS Tjandra JJ Green MD. Morbidities of adjuvant chemotherapy and radiotherapy for resectable rectal cancer: an overview. Dis Colon Rectum. (1999) 42:403–18. doi: 10.1007/bf02236362 10223765

[45] Morris MC Winer LK Lee TC Shah SA Rafferty JF Paquette IM. Omission of adjuvant chemotherapy in rectal cancer patients with pathologic complete response: a national analysis. J Gastrointest Surg. (2021) 25:1857–65. doi: 10.1007/s11605-020-04749-6 PMC7388436 32728821

[46] Manzini G Hapke F Hines IN Henne-Bruns D Kremer M. Adjuvant chemotherapy in curatively resected rectal cancer: How valid are the data? World J Gastrointest Oncol. (2020) 12:503–13. doi: 10.4251/wjgo.v12.i4.503 PMC7191332 32368327

[47] Chao MW Tjandra JJ Gibbs P McLaughlin S. How safe is adjuvant chemotherapy and radiotherapy for rectal cancer? Asian J Surg. (2004) 27:147–61. doi: 10.1016/s1015-9584(09)60331-6 15140670

[48] Rahma OE Yothers G Hong TS Russell MM You YN Parker W. Use of total neoadjuvant therapy for locally advanced rectal cancer: initial results from the pembrolizumab arm of a phase 2 randomized clinical trial. JAMA Oncol. (2021) 7:1225–30. doi: 10.1001/jamaoncol.2021.1683 PMC8251652 34196693

[49] Petrelli F Trevisan F Cabiddu M Sgroi G Bruschieri L Rausa E. Total neoadjuvant therapy in rectal cancer: A systematic review and meta-analysis of treatment outcomes. Ann Surg. (2020) 271:440–8. doi: 10.1097/sla.0000000000003471 31318794

[50] Lo Greco MC La Rocca M Marano G Finocchiaro I Liardo RLE Milazzotto R. Integrated intensified chemoradiation in the setting of total neoadjuvant therapy (TNT) in patients with locally advanced rectal cancer: A retrospective single-arm study on feasibility and efficacy. Cancers (Basel). (2023) 15. doi: 10.3390/cancers15030921 PMC9913523 36765878

[51] Kennecke HF Bahnson HT Lin B O’Rourke C Kaplan J Pham H. Patterns of practice and improvements in survival among patients with stage 2/3 rectal cancer treated with trimodality therapy. JAMA Oncol. (2022) 8:1466–70. doi: 10.1001/jamaoncol.2022.2831 PMC9389431 35980607

[52] Kasi A Abbasi S Handa S Al-Rajabi R Saeed A Baranda J. Total neoadjuvant therapy vs standard therapy in locally advanced rectal cancer: A systematic review and meta-analysis. JAMA Netw Open. (2020) 3:e2030097. doi: 10.1001/jamanetworkopen.2020.30097 33326026 PMC7745099

[53] Yang R Wu T Yu J Cai X Li G Li X. Locally advanced rectal cancer with dMMR/MSI-H may be excused from surgery after neoadjuvant anti-PD-1 monotherapy: a multiple-center, cohort study. Front Immunol. (2023) 14:1182299. doi: 10.3389/fimmu.2023.1182299 37441082 PMC10333582

[54] Zhou L Yang XQ Zhao GY Wang FJ Liu X. Meta-analysis of neoadjuvant immunotherapy for non-metastatic colorectal cancer. Front Immunol. (2023) 14:1044353. doi: 10.3389/fimmu.2023.1044353 36776899 PMC9911889

[55] Marolleau P Tougeron D Allignet B Cohen R Sefrioui D Gallet B. Complete pathological response after chemotherapy or immune checkpoint inhibitors in deficient MMR metastatic colorectal cancer: Results of a retrospective multicenter study. Int J Cancer. (2023) 153:1376–85. doi: 10.1002/ijc.34636 37403609

[56] Kasi PM Hidalgo M Jafari MD Yeo H Lowenfeld L Khan U. Neoadjuvant botensilimab plus balstilimab response pattern in locally advanced mismatch repair proficient colorectal cancer. Oncogene. (2023) 42:3252–9. doi: 10.1038/s41388-023-02835-y PMC10611560 37731056

[57] Fokas E Appelt A Glynne-Jones R Beets G Perez R Garcia-Aguilar J. International consensus recommendations on key outcome measures for organ preservation after (chemo)radiotherapy in patients with rectal cancer. Nat Rev Clin Oncol. (2021) 18:805–16. doi: 10.1038/s41571-021-00538-5 34349247

[58] Wynn GR Bhasin N Macklin CP George ML. Complete clinical response to neoadjuvant chemoradiotherapy in patients with rectal cancer: opinions of British and Irish specialists. Colorectal Dis. (2010) 12:327–33. doi: 10.1111/j.1463-1318.2009.01962.x 19555388

[59] Hiotis SP Weber SM Cohen AM Minsky BD Paty PB Guillem JG. Assessing the predictive value of clinical complete response to neoadjuvant therapy for rectal cancer: an analysis of 488 patients. J Am Coll Surg. (2002) 194:131–5; discussion 5-6. doi: 10.1016/s1072-7515(01)01159-0 11848629

[60] van der Valk MJM Hilling DE Bastiaannet E Meershoek-Klein Kranenbarg E Beets GL Figueiredo NL. Long-term outcomes of clinical complete responders after neoadjuvant treatment for rectal cancer in the International Watch & Wait Database (IWWD): an international multicentre registry study. Lancet. (2018) 391:2537–45. doi: 10.1016/s0140-6736(18)31078-x 29976470

[61] Nilsson PJ Ahlberg M Kordnejad S Holm T Martling A. Organ preservation following short-course radiotherapy for rectal cancer. BJS Open. (2021) 5. doi: 10.1093/bjsopen/zrab093 PMC8536864 34686879

[62] Jimenez-Rodriguez RM Quezada-Diaz F Hameed I Kalabin A Patil S Smith JJ. Organ preservation in patients with rectal cancer treated with total neoadjuvant therapy. Dis Colon Rectum. (2021) 64:1463–70. doi: 10.1097/dcr.0000000000002122 PMC8820240 34508014

[63] Hsu YJ Chern YJ Lai IL Chiang SF Liao CK Tsai WS. Usefulness of close surveillance for rectal cancer patients after neoadjuvant chemoradiotherapy. Open Med (Wars). (2022) 17:1438–48. doi: 10.1515/med-2022-0555 PMC9449684 36128450

[64] Dizdarevic E Frøstrup Hansen T Pløen J Henrik Jensen L Lindebjerg J Rafaelsen S. Long-term patient-reported outcomes after high-dose chemoradiation therapy for nonsurgical management of distal rectal cancer. Int J Radiat Oncol Biol Phys. (2020) 106:556–63. doi: 10.1016/j.ijrobp.2019.10.046 31707122

[65] Garant A Florianova L Gologan A Spatz A Faria J Morin N. Do clinical criteria reflect pathologic complete response in rectal cancer following neoadjuvant therapy? Int J Colorectal Dis. (2018) 33:727–33. doi: 10.1007/s00384-018-3033-7 29602976

[66] Smith JJ Chow OS Gollub MJ Nash GM Temple LK Weiser MR. Organ Preservation in Rectal Adenocarcinoma: a phase II randomized controlled trial evaluating 3-year disease-free survival in patients with locally advanced rectal cancer treated with chemoradiation plus induction or consolidation chemotherapy, and total mesorectal excision or nonoperative management. BMC Cancer. (2015) 15:767. doi: 10.1186/s12885-015-1632-z 26497495 PMC4619249

[67] Chinese Watch & Wait Database Research Cooperation Group(CWWD); Chinese Association of Surgeons, Chinese Society of Coloproctology, Chinese Medical Doctor Association; Chinese Society of Colorectal Surgery, Chinese Medical Association; Colorectal Cancer Physician Specialty Committee, Chinese Medical Doctor Association; Radiation Therapy Specialty Committee, Chinese Anticancer Association. Consensus on the Watch and Wait policy in rectal cancer patients after neoadjuvant treatment (2020 version). Zhonghua Wei Chang Wai Ke Za Zhi. (2020) 23:1–9. doi: 10.3760/cma.j.issn.1671-0274.2020.01.001 31958923

[68] Hupkens BJP Maas M Martens MH van der Sande ME Lambregts DMJ Breukink SO. Organ preservation in rectal cancer after chemoradiation: should we extend the observation period in patients with a clinical near-complete response? Ann Surg Oncol. (2018) 25:197–203. doi: 10.1245/s10434-017-6213-8 29134378

[69] Cotte E Passot G Decullier E Maurice C Glehen O François Y. Pathologic response, when increased by longer interval, is a marker but not the cause of good prognosis in rectal cancer: 17-year follow-up of the lyon R90-01 randomized trial. Int J Radiat Oncol Biol Phys. (2016) 94:544–53. doi: 10.1016/j.ijrobp.2015.10.061 26723110

[70] Bujko K Nowacki MP Nasierowska-Guttmejer A Michalski W Bebenek M Pudełko M. Sphincter preservation following preoperative radiotherapy for rectal cancer: report of a randomised trial comparing short-term radiotherapy vs. conventionally fractionated radiochemotherapy. Radiother Oncol. (2004) 72:15–24. doi: 10.1016/j.radonc.2003.12.006 15236870

[71] Fokas E Allgäuer M Polat B Klautke G Grabenbauer GG Fietkau R. Randomized phase II trial of chemoradiotherapy plus induction or consolidation chemotherapy as total neoadjuvant therapy for locally advanced rectal cancer: CAO/ARO/AIO-12. J Clin Oncol. (2019) 37:3212–22. doi: 10.1200/jco.19.00308 31150315

[72] Fokas E Schlenska-Lange A Polat B Klautke G Grabenbauer GG Fietkau R. Chemoradiotherapy plus induction or consolidation chemotherapy as total neoadjuvant therapy for patients with locally advanced rectal cancer: long-term results of the CAO/ARO/AIO-12 randomized clinical trial. JAMA Oncol. (2022) 8:e215445. doi: 10.1001/jamaoncol.2021.5445 34792531 PMC8603234

[73] Hayden DM Jakate S Pinzon MC Giusto D Francescatti AB Brand MI. Tumor scatter after neoadjuvant therapy for rectal cancer: are we dealing with an invisible margin? Dis Colon Rectum. (2012) 55:1206–12. doi: 10.1097/DCR.0b013e318269fdb3 23135577

[74] Chen J Wu Z Zhang X Liu Z Wang Y Shan F. Optimized tools and timing of response reassessment after neoadjuvant chemoradiation in rectal cancer. Int J Colorectal Dis. (2022) 37:2321–33. doi: 10.1007/s00384-022-04268-7 PMC9569175 36243807

[75] Duldulao MP Lee W Streja L Chu P Li W Chen Z. Distribution of residual cancer cells in the bowel wall after neoadjuvant chemoradiation in patients with rectal cancer. Dis Colon Rectum. (2013) 56:142–9. doi: 10.1097/DCR.0b013e31827541e2 PMC4674069 23303141

[76] Perez RO Habr-Gama A Pereira GV Lynn PB Alves PA Proscurshim I. Role of biopsies in patients with residual rectal cancer following neoadjuvant chemoradiation after downsizing: can they rule out persisting cancer? Colorectal Dis. (2012) 14:714–20. doi: 10.1111/j.1463-1318.2011.02761.x 22568644

[77] Han JG Sun LT Zhai ZW Xia PD Hu H Zhang D. The value of transanal multipoint full-layer puncture biopsy in determining the response degree of rectal cancer following neoadjuvant therapy: a prospective multicenter study. Zhonghua Wai Ke Za Zhi. (2023) 61:768–74. doi: 10.3760/cma.j.cn112139-20230417-00170 37491169

[78] Xiao Y Xue H Zhong G Zhou W Xu L Du X. Predictive value of preoperative imaging and postoperative pathology on clinical complete response after neoadjuvant chemoradiation for locally advanced rectal cancer. Zhonghua Wei Chang Wai Ke Za Zhi. (2015) 18:474–7. doi: 10.3760/cma.j.issn.1671-0274.2015.05.018 26013867

[79] Safatle-Ribeiro AV Ribeiro U Jr. Lata J Baba ER Lenz L da Costa Martins B. The role of probe-based confocal laser endomicroscopy (pCLE) in the diagnosis of sustained clinical complete response under watch-and-wait strategy after neoadjuvant chemoradiotherapy for locally advanced rectal adenocarcinoma: a score validation. J Gastrointest Surg. (2023) 27:1903–12. doi: 10.1007/s11605-023-05732-7 37291428

[80] Safatle-Ribeiro AV Marques CFS Pires C Arraes L Baba ER Meirelles L. Diagnosis of clinical complete response by probe-based confocal laser endomicroscopy (pCLE) after chemoradiation for advanced rectal cancer. J Gastrointest Surg. (2021) 25:357–68. doi: 10.1007/s11605-020-04878-y 33443686

[81] Battersby NJ Dattani M Rao S Cunningham D Tait D Adams R. A rectal cancer feasibility study with an embedded phase III trial design assessing magnetic resonance tumour regression grade (mrTRG) as a novel biomarker to stratify management by good and poor response to chemoradiotherapy (TRIGGER): study protocol for a randomised controlled trial. Trials. (2017) 18:394. doi: 10.1186/s13063-017-2085-2 28851403 PMC5576102

[82] Yoen H Park HE Kim SH Yoon JH Hur BY Bae JS. Prognostic value of tumor regression grade on MR in rectal cancer: A large-scale, single-center experience. Korean J Radiol. (2020) 21:1065–76. doi: 10.3348/kjr.2019.0797 PMC7371618 32691542

[83] Yang IJ Suh JW Lee J Ahn HM Oh HK Kim DW. Comparison of tumor regression grade and clinical stage based on MRI image as a selection criterion for non-radical management after concurrent chemoradiotherapy in locally advanced rectal cancer: a multicenter, retrospective, cross-sectional study. Int J Colorectal Dis. (2022) 37:1561–8. doi: 10.1007/s00384-022-04193-9 35648208

[84] Suzuki C Halperin SK Nilsson PJ Martling A Holm T. Initial magnetic resonance imaging tumour regression grade (mrTRG) as response evaluation after neoadjuvant treatment predicts sustained complete response in patients with rectal cancer. Eur J Surg Oncol. (2022) 48:1643–9. doi: 10.1016/j.ejso.2022.02.012 35272899

[85] Jang JK Lee JL Park SH Park HJ Park IJ Kim JH. Magnetic resonance tumour regression grade and pathological correlates in patients with rectal cancer. Br J Surg. (2018) 105:1671–9. doi: 10.1002/bjs.10898 29893988

[86] Jang JK Choi SH Park SH Kim KW Kim HJ Lee JS. MR tumor regression grade for pathological complete response in rectal cancer post neoadjuvant chemoradiotherapy: a systematic review and meta-analysis for accuracy. Eur Radiol. (2020) 30:2312–23. doi: 10.1007/s00330-019-06565-2 31953656

[87] Judge SJ Malekzadeh P Corines MJ Gollub MJ Horvat N Gonen M. Watch and wait in rectal cancer patients with residual mucin on magnetic resonance imaging following neoadjuvant therapy. J Natl Cancer Inst. (2024) 116:1761–6. doi: 10.1093/jnci/djae152 PMC11542991 38937278

[88] Xu N Huang FC Li WL Luan X Jiang YM He B. Predictive value of combination of MRI tumor regression grade and apparent diffusion coefficient for pathological complete remission after neoadjuvant treatment of locally advanced rectal cancer. Zhonghua Wei Chang Wai Ke Za Zhi. (2021) 24:359–65. doi: 10.3760/cma.j.cn.441530-20200225-00089 33878826

[89] Ince S Itani M Henke LE Smith RK Wise PE Mutch MG. FDG-PET/MRI for nonoperative management of rectal cancer: A prospective pilot study. Tomography. (2022) 8:2723–34. doi: 10.3390/tomography8060227 PMC9680346 36412686

[90] Hötker AM Tarlinton L Mazaheri Y Woo KM Gönen M Saltz LB. Multiparametric MRI in the assessment of response of rectal cancer to neoadjuvant chemoradiotherapy: A comparison of morphological, volumetric and functional MRI parameters. Eur Radiol. (2016) 26:4303–12. doi: 10.1007/s00330-016-4283-9 PMC5203699 26945761

[91] Perez RO Habr-Gama A São Julião GP Lynn PB Sabbagh C Proscurshim I. Predicting complete response to neoadjuvant CRT for distal rectal cancer using sequential PET/CT imaging. Tech Coloproctol. (2014) 18:699–708. doi: 10.1007/s10151-013-1113-9 24509716

[92] Dos Anjos DA Perez RO Habr-Gama A São Julião GP Vailati BB Fernandez LM. Semiquantitative volumetry by sequential PET/CT may improve prediction of complete response to neoadjuvant chemoradiation in patients with distal rectal cancer. Dis Colon Rectum. (2016) 59:805–12. doi: 10.1097/dcr.0000000000000655 27505108

[93] McMahon RK O’Cathail SM Nair H Steele CW Platt JJ Digby M. The neoadjuvant rectal score and a novel magnetic resonance imaging based neoadjuvant rectal score are stage independent predictors of long-term outcome in locally advanced rectal cancer. Colorectal Dis. (2023) 25:1783–94. doi: 10.1111/codi.16667 37485654

[94] Smith FM Wiland H Mace A Pai RK Kalady MF. Clinical criteria underestimate complete pathological response in rectal cancer treated with neoadjuvant chemoradiotherapy. Dis Colon Rectum. (2014) 57:311–5. doi: 10.1097/DCR.0b013e3182a84eba 24509452

[95] Crimì F Spolverato G Lacognata C Garieri M Cecchin D Urso ED. 18F-FDG PET/MRI for rectal cancer TNM restaging after preoperative chemoradiotherapy: initial experience. Dis Colon Rectum. (2020) 63:310–8. doi: 10.1097/dcr.0000000000001568 31842163

[96] Sclafani F Brown G Cunningham D Wotherspoon A Mendes LST Balyasnikova S. Comparison between MRI and pathology in the assessment of tumour regression grade in rectal cancer. Br J Cancer. (2017) 117:1478–85. doi: 10.1038/bjc.2017.320 PMC5680467 28934761

[97] Wang A Zhou J Wang G Zhang B Xin H Zhou H. Deep learning of endoscopic features for the assessment of neoadjuvant therapy response in locally advanced rectal cancer. Asian J Surg. (2023) 46:3568–74. doi: 10.1016/j.asjsur.2023.03.165 37062601

[98] Chen X Chen J He X Xu L Liu W Lin D. Endoscopy-based deep convolutional neural network predicts response to neoadjuvant treatment for locally advanced rectal cancer. Front Physiol. (2022) 13:880981. doi: 10.3389/fphys.2022.880981 35574447 PMC9091815

[99] Chang L Zhang X He L Ma Q Fang T Jiang C. Prognostic value of ctDNA detection in patients with locally advanced rectal cancer undergoing neoadjuvant chemoradiotherapy: A systematic review and meta-analysis. Oncologist. (2023) 28(12):e1198–208. doi: 10.1093/oncolo/oyad151 37294663 PMC10712909

[100] Morais M Pinto DM MaChado JC Carneiro S. ctDNA on liquid biopsy for predicting response and prognosis in locally advanced rectal cancer: A systematic review. Eur J Surg Oncol. (2022) 48:218–27. doi: 10.1016/j.ejso.2021.08.034 34511270

[101] Morais M Fonseca T Melo-Pinto D Prieto I Vilares AT Duarte AL. Evaluation of ctDNA in the prediction of response to neoadjuvant therapy and prognosis in locally advanced rectal cancer patients: A prospective study. Pharm (Basel). (2023) 16. doi: 10.3390/ph16030427 PMC10057108 36986526

[102] MaChado Carvalho JV Dutoit V Corrò C Koessler T. Promises and challenges of predictive blood biomarkers for locally advanced rectal cancer treated with neoadjuvant chemoradiotherapy. Cells. (2023) 12. doi: 10.3390/cells12030413 PMC9913546 36766755

[103] Wang Y Yang L Bao H Fan X Xia F Wan J. Utility of ctDNA in predicting response to neoadjuvant chemoradiotherapy and prognosis assessment in locally advanced rectal cancer: A prospective cohort study. PloS Med. (2021) 18:e1003741. doi: 10.1371/journal.pmed.1003741 34464382 PMC8407540

[104] Liu WY Zhang W Tang Y Chen SL Li N Lei JQ. Metastasis risk stratification and response prediction through dynamic viable circulating tumor cell counts for rectal cancer in a neoadjuvant setting. Cancer Med. (2023) 12:11438–50. doi: 10.1002/cam4.5860 PMC10242869 37014817

[105] Silva VSE Abdallah EA Flores BCT Braun AC Costa DJF Ruano APC. Molecular and dynamic evaluation of proteins related to resistance to neoadjuvant treatment with chemoradiotherapy in circulating tumor cells of patients with locally advanced rectal cancer. Cells. (2021) 10. doi: 10.3390/cells10061539 PMC8234587 34207124

[106] Troncarelli Flores BC Souza ESV Ali Abdallah E Mello CAL Gobo Silva ML Gomes Mendes G. Molecular and kinetic analyses of circulating tumor cells as predictive markers of treatment response in locally advanced rectal cancer patients. Cells. (2019) 8. doi: 10.3390/cells8070641 PMC6679115 31247977

[107] Eastern Rectal Cancer Response Collaborative, Ireland. A multicentre cohort study assessing the utility of routine blood tests as adjuncts to identify complete responders in rectal cancer following neoadjuvant chemoradiotherapy. Int J Colorectal Dis. (2022) 37:957–65. doi: 10.1007/s00384-022-04103-z PMC8976819 35325271

[108] Dou X Wang R Meng X Yan H Jiang S Zhu K. The prognostic role of TCF4 expression in locally advanced rectal cancer patients treated with neoadjuvant chemoradiotherapy. Cancer biomark. (2015) 15:181–8. doi: 10.3233/cbm-140452 PMC12928528 25519018

[109] Dou X Wang RB Meng XJ Yan HJ Jiang SM Zhu KL. PDCD4 as a predictor of sensitivity to neoadjuvant chemoradiotherapy in locally advanced rectal cancer patients. Asian Pac J Cancer Prev. (2014) 15:825–30. doi: 10.7314/apjcp.2014.15.2.825 24568503

[110] Dou X Wang RB Yan HJ Jiang SM Meng XJ Zhu KL. Circulating lymphocytes as predictors of sensitivity to preoperative chemoradiotherapy in rectal cancer cases. Asian Pac J Cancer Prev. (2013) 14:3881–5. doi: 10.7314/apjcp.2013.14.6.3881 23886201

[111] Nassar A Aly NE Jin Z Aly EH. ctDNA as a predictor of outcome after curative resection for locally advanced rectal cancer: systematic review and meta-analysis. Colorectal Dis. (2024) 26:1346–58. doi: 10.1111/codi.17039 38802990

[112] Tsai KY Huang PS Chu PY Nguyen TNA Hung HY Hsieh CH. Current applications and future directions of circulating tumor cells in colorectal cancer recurrence. Cancers (Basel). (2024) 16. doi: 10.3390/cancers16132316 PMC11240518 39001379

[113] Wang XH Zhou CJ Zhang S Wang QX Xiao WW Ding PR. Comparison of long-term efficacy between watch and wait strategy and total mesorectal excision in locally advanced rectal cancer patients with clinical complete response after neoadjuvant therapy. Zhonghua Wei Chang Wai Ke Za Zhi. (2020) 23:266–73. doi: 10.3760/cma.j.cn.441530-20200224-00081 32192306

[114] Capelli G De Simone I Spolverato G Cinquini M Moschetti I Lonardi S. Non-operative management versus total mesorectal excision for locally advanced rectal cancer with clinical complete response after neoadjuvant chemoradiotherapy: a GRADE approach by the rectal cancer guidelines writing group of the italian association of medical oncology (AIOM). J Gastrointest Surg. (2020) 24:2150–9. doi: 10.1007/s11605-020-04635-1 32394125

[115] Xu F Li H Guo C Yang Z Gao J Zhang X. Incidence and risk factors of surgical complications and anastomotic leakage after transanal total mesorectal excision for middle and low rectal cancer. J Gastrointest Surg. (2023) 27:373–81. doi: 10.1007/s11605-022-05546-z 36538254

[116] Sun R Dai Z Zhang Y Lu J Zhang Y Xiao Y. The incidence and risk factors of low anterior resection syndrome (LARS) after sphincter-preserving surgery of rectal cancer: a systematic review and meta-analysis. Support Care Cancer. (2021) 29:7249–58. doi: 10.1007/s00520-021-06326-2 34296335

[117] Pape E Pattyn P Van Hecke A Somers N Van de Putte D Ceelen W. Impact of low anterior resection syndrome (LARS) on the quality of life and treatment options of LARS - A cross sectional study. Eur J Oncol Nurs. (2021) 50:101878. doi: 10.1016/j.ejon.2020.101878 33246248

[118] van Heinsbergen M Janssen-Heijnen ML Leijtens JW Slooter GD Konsten JL. Bowel dysfunction after sigmoid resection underestimated: Multicentre study on quality of life after surgery for carcinoma of the rectum and sigmoid. Eur J Surg Oncol. (2018) 44:1261–7. doi: 10.1016/j.ejso.2018.05.003 29778617

[119] Hüser N Michalski CW Erkan M Schuster T Rosenberg R Kleeff J. Systematic review and meta-analysis of the role of defunctioning stoma in low rectal cancer surgery. Ann Surg. (2008) 248:52–60. doi: 10.1097/SLA.0b013e318176bf65 18580207

[120] Kneist W Hanke L Kauff DW Lang H. Surgeons’ assessment of internal anal sphincter nerve supply during TaTME - inbetween expectations and reality. Minim Invasive Ther Allied Technol. (2016) 25:241–6. doi: 10.1080/13645706.2016.1197269 PMC5044775 27333465

[121] Young DO Kumar AS. Local excision of rectal cancer. Surg Clin North Am. (2017) 97:573–85. doi: 10.1016/j.suc.2017.01.007 28501248

[122] Smart CJ Korsgen S Hill J Speake D Levy B Steward M. Multicentre study of short-course radiotherapy and transanal endoscopic microsurgery for early rectal cancer. Br J Surg. (2016) 103:1069–75. doi: 10.1002/bjs.10171 27146472

[123] Pucciarelli S De Paoli A Guerrieri M La Torre G Maretto I De Marchi F. Local excision after preoperative chemoradiotherapy for rectal cancer: results of a multicenter phase II clinical trial. Dis Colon Rectum. (2013) 56:1349–56. doi: 10.1097/DCR.0b013e3182a2303e 24201388

[124] Stijns RCH de Graaf EJR Punt CJA Nagtegaal ID Nuyttens J van Meerten E. Long-term oncological and functional outcomes of chemoradiotherapy followed by organ-sparing transanal endoscopic microsurgery for distal rectal cancer: the CARTS study. JAMA Surg. (2019) 154:47–54. doi: 10.1001/jamasurg.2018.3752 30304338 PMC6439861

[125] Rullier E Rouanet P Tuech JJ Valverde A Lelong B Rivoire M. Organ preservation for rectal cancer (GRECCAR 2): a prospective, randomised, open-label, multicentre, phase 3 trial. Lancet. (2017) 390:469–79. doi: 10.1016/s0140-6736(17)31056-5 28601342

[126] Teste B Rouanet P Tuech JJ Valverde A Lelong B Rivoire M. Early and late morbidity of local excision after chemoradiotherapy for rectal cancer. BJS Open. (2021) 5. doi: 10.1093/bjsopen/zrab043 PMC8183183 34097005

[127] Marchegiani F Palatucci V Capelli G Guerrieri M Belluco C Rega D. Rectal sparing approach after neoadjuvant therapy in patients with rectal cancer: the preliminary results of the reSARCh trial. Ann Surg Oncol. (2022) 29:1880–9. doi: 10.1245/s10434-021-11121-8 34855063

[128] Boubaddi M Fleming C Vendrely V Frulio N Salut C Rullier E. Feasibility study of a Response Surveillance Program in locally advanced mid and low rectal cancer to increase organ preservation. Eur J Surg Oncol. (2023) 49:237–43. doi: 10.1016/j.ejso.2022.08.031 36114048

[129] Perez RO Habr-Gama A Lynn PB São Julião GP Bianchi R Proscurshim I. Transanal endoscopic microsurgery for residual rectal cancer (ypT0-2) following neoadjuvant chemoradiation therapy: another word of caution. Dis Colon Rectum. (2013) 56:6–13. doi: 10.1097/DCR.0b013e318273f56f 23222274

[130] Cerdan-Santacruz C São Julião GP Vailati BB Corbi L Habr-Gama A Perez RO. Watch and wait approach for rectal cancer. J Clin Med. (2023) 12. doi: 10.3390/jcm12082873 PMC10143332 37109210

[131] Zhang X Ding R Li J Wu T Shen Z Li S. Efficacy and safety of the “watch-and-wait” approach for rectal cancer with clinical complete response after neoadjuvant chemoradiotherapy: a meta-analysis. Surg Endosc. (2022) 36:2233–44. doi: 10.1007/s00464-021-08932-x 34981233

[132] Tan S Gao Q Cui Y Ou Y Huang S Feng W. Oncologic outcomes of watch-and-wait strategy or surgery for low to intermediate rectal cancer in clinical complete remission after adjuvant chemotherapy: a systematic review and meta-analysis. Int J Colorectal Dis. (2023) 38:246. doi: 10.1007/s00384-023-04534-2 37787779

[133] Jankowski M Pietrzak L Rupiński M Michalski W Hołdakowska A Paciorek K. Watch-and-wait strategy in rectal cancer: Is there a tumour size limit? Results from two pooled prospective studies. Radiother Oncol. (2021) 160:229–35. doi: 10.1016/j.radonc.2021.05.014 34023328

[134] Huang CM Huang MY Huang CW Tsai HL Su WC Chang WC. Machine learning for predicting pathological complete response in patients with locally advanced rectal cancer after neoadjuvant chemoradiotherapy. Sci Rep. (2020) 10:12555. doi: 10.1038/s41598-020-69345-9 32724164 PMC7387337

[135] Garland ML Vather R Bunkley N Pearse M Bissett IP. Clinical tumour size and nodal status predict pathologic complete response following neoadjuvant chemoradiotherapy for rectal cancer. Int J Colorectal Dis. (2014) 29:301–7. doi: 10.1007/s00384-013-1821-7 24420737

[136] Habr-Gama A São Julião GP Gama-Rodrigues J Vailati BB Ortega C Fernandez LM. Baseline T classification predicts early tumor regrowth after nonoperative management in distal rectal cancer after extended neoadjuvant chemoradiation and initial complete clinical response. Dis Colon Rectum. (2017) 60:586–94. doi: 10.1097/dcr.0000000000000830 28481852

[137] Guido A Cuicchi D Castellucci P Cellini F Di Fabio F Llimpe FLR. Adaptive Individualized high-dose preoperAtive (AIDA) chemoradiation in high-risk rectal cancer: a phase II trial. Eur J Nucl Med Mol Imaging. (2023) 50:572–80. doi: 10.1007/s00259-022-05944-0 PMC9816267 36127416

[138] Prata I Eriksson M Krdzalic J Kranenbarg EM Roodvoets AGH Beets-Tan R. Results of a diagnostic imaging audit in a randomised clinical trial in rectal cancer highlight the importance of careful planning and quality control. Insights Imaging. (2023) 14:206. doi: 10.1186/s13244-023-01552-0 38001376 PMC10673763

[139] He F Ju HQ Ding Y Jiang Z Li Z Huang B. Association between adjuvant chemotherapy and survival in patients with rectal cancer and pathological complete response after neoadjuvant chemoradiotherapy and resection. Br J Cancer. (2020) 123:1244–52. doi: 10.1038/s41416-020-0989-1 PMC7553967 32724220

[140] Lin W Wee IJY Seow-En I Chok AY Tan EK. Survival outcomes of salvage surgery in the watch-and-wait approach for rectal cancer with clinical complete response after neoadjuvant chemoradiotherapy: a systematic review and meta-analysis. Ann Coloproctol. (2023) 39:447–56. doi: 10.3393/ac.2022.01221.0174 PMC10781598 38185947

[141] Han NY Kim MJ Park BJ Sung DJ. Location of rectal cancer as determined using rectal magnetic resonance imaging, and its relationship with pulmonary metastasis. Turk J Gastroenterol. (2014) 25:661–8. doi: 10.5152/tjg.2014.5616 25599778

[142] Akiyoshi T Yamaguchi T Hiratsuka M Mukai T Hiyoshi Y Nagasaki T. Oncologic impact of lateral lymph node metastasis at the distal lateral compartment in locally advanced low rectal cancer after neoadjuvant (chemo)radiotherapy. Eur J Surg Oncol. (2021) 47:3157–65. doi: 10.1016/j.ejso.2021.07.011 34284904

[143] Fernandez LM São Julião GP Figueiredo NL Beets GL van der Valk MJM Bahadoer RR. Conditional recurrence-free survival of clinical complete responders managed by watch and wait after neoadjuvant chemoradiotherapy for rectal cancer in the International Watch & Wait Database: a retrospective, international, multicentre registry study. Lancet Oncol. (2021) 22:43–50. doi: 10.1016/s1470-2045(20)30557-x 33316218

[144] Rega D Granata V Romano C Fusco R Aversano A Ravo V. Total mesorectal excision after rectal-sparing approach in locally advanced rectal cancer patients after neoadjuvant treatment: a high volume center experience. Ther Adv Gastrointest Endosc. (2024) 17:26317745241231098. doi: 10.1177/26317745241231098 39044726 PMC11265235

[145] Dattani M Heald RJ Goussous G Broadhurst J São Julião GP Habr-Gama A. Oncological and survival outcomes in watch and wait patients with a clinical complete response after neoadjuvant chemoradiotherapy for rectal cancer: A systematic review and pooled analysis. Ann Surg. (2018) 268:955–67. doi: 10.1097/sla.0000000000002761 29746338

[146] Fernandez LM São Julião GP Renehan AG Beets GL Papoila AL Vailati BB. The risk of distant metastases in patients with clinical complete response managed by watch and wait after neoadjuvant therapy for rectal cancer: the influence of local regrowth in the international watch and wait database. Dis Colon Rectum. (2023) 66:41–9. doi: 10.1097/dcr.0000000000002494 36515514

[147] Wang L Li S Zhang X Sun T Du C Chen N. Long-term prognostic analysis on complete/near-complete clinical remission for mid-low rectal cancer after neoadjuvant chemoradiotherapy. Zhonghua Wei Chang Wai Ke Za Zhi. (2018) 21:1240–8. doi: 10.3760/cma.j.issn.1671-0274.2018.11.008 30506534

[148] Lee YT. Local and regional recurrence of carcinoma of the colon and rectum: II. Factors relating to operative technique. Surg Oncol. (1996) 5:1–13. doi: 10.1016/s0960-7404(96)80016-8 8837299

[149] Socha J Kępka L Michalski W Paciorek K Bujko K. The risk of distant metastases in rectal cancer managed by a watch-and-wait strategy - A systematic review and meta-analysis. Radiother Oncol. (2020) 144:1–6. doi: 10.1016/j.radonc.2019.10.009 31710938

[150] Ryan ÉJ O’Sullivan DP Kelly ME Syed AZ Neary PC O’Connell PR. Meta-analysis of the effect of extending the interval after long-course chemoradiotherapy before surgery in locally advanced rectal cancer. Br J Surg. (2019) 106:1298–310. doi: 10.1002/bjs.11220 31216064

[151] Samee A Selvasekar CR. Current trends in staging rectal cancer. World J Gastroenterol. (2011) 17:828–34. doi: 10.3748/wjg.v17.i7.828 PMC3051133 21412492

[152] Labiad C Alric H Barret M Cazelles A Rahmi G Karoui M. Management after local excision of small rectal cancers. Indications for completion total mesorectal excision and possible alternatives. J Visc Surg. (2024) 161(3):173–81. doi: 10.1016/j.jviscsurg.2024.02.003 38448362

[153] Wyatt J Powell SG Ahmed S. Watch and wait in rectal cancer after a complete response to chemoradiotherapy &x2013; is it safe and are we doing enough? Clin Oncol. (2023) 35:117–23. doi: 10.1016/j.clon.2022.10.004

[154] Glynne-Jones R Wyrwicz L Tiret E Brown G Rödel C Cervantes A. Rectal cancer: ESMO Clinical Practice Guidelines for diagnosis, treatment and follow-up^&x2020;^ . Ann Oncol. (2017) 28:iv22–40. doi: 10.1093/annonc/mdx224 28881920

